# The Effect of Added Ligands on the Reactions of [Ni(COD)(dppf)] with Alkyl Halides: Halide Abstraction May Be Reversible

**DOI:** 10.1021/acs.organomet.1c00280

**Published:** 2021-06-16

**Authors:** Megan E. Greaves, Thomas O. Ronson, Feliu Maseras, David J. Nelson

**Affiliations:** † WestCHEM Department of Pure and Applied Chemistry, University of Strathclyde, 295 Cathedral Street, Glasgow G1 1XL, Scotland; ‡ Chemical Development, Pharmaceutical Technology and Development, Operations, AstraZeneca, Macclesfield SK10 2NA, U.K.; § Institute of Chemical Research of Catalonia (ICIQ), The Barcelona Institute of Science and Technology, Av. Països Catalans 16, Tarragona 43007, Spain

## Abstract

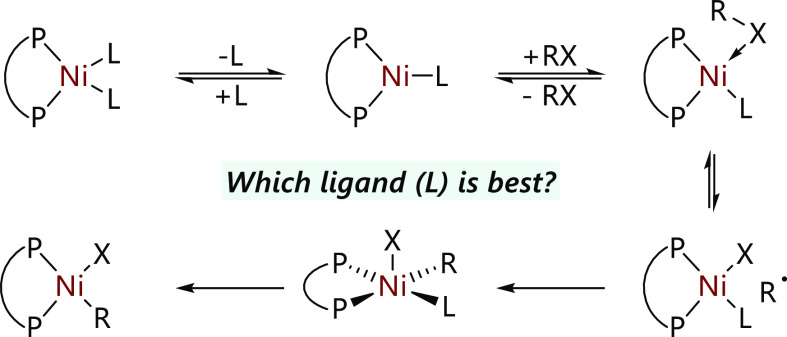

The reactions of dppf-nickel(0) with alkyl halides proceed via three-coordinate nickel(0) intermediates of the form [Ni(dppf)(L)]. The effects of the identity of the added ligand (L) on catalyst speciation and the rates of reactions of [Ni(COD)(dppf)] with alkyl halides have been investigated using kinetic experiments and density functional theory calculations. A series of monodentate ligands have been investigated in attempts to identify trends in reactivity. Sterically bulky and electron-donating ligands are found to decrease the reaction rate. It was found that (i) the halide abstraction step is not always irreversible and the subsequent recombination of a nickel(I) complex with an alkyl halide can have a significant effect on the overall rate of the reaction and (ii) some ligands lead to very stable [Ni(dppf)(L)_2_] species. The yields of prototypical (dppf)nickel-catalyzed Kumada cross-coupling reactions of alkyl halides are significantly improved by the addition of free ligands, which provides another important variable to consider when optimizing nickel-catalyzed reactions of alkyl halides.

## Introduction

The importance of molecules that contain a large number of sp^3^ centers in industries such as pharmaceuticals and agrochemicals is driving much of the reaction discovery and development in the field of nickel catalysis.^1,2^ However, despite recent advances in our mechanistic understanding of nickel catalysis,^3,4^ gaps in this understanding still remain. This is apparent for the case of the reactions of nickel(0) complexes with alkyl halides, which are quite different from the reactions of nickel(0) complexes with sp^2^ organohalides.^5^ The reactions of alkyl halides have a greater propensity to involve radical intermediates, and deleterious β-hydride elimination presents further challenges. We have recently focused our attention on the reactions of alkyl halides with nickel(0), with the aim of developing a better understanding of these reactions and thereby underpinning future reaction discovery, development, and understanding.

The outcomes of nickel-catalyzed reactions can be extremely sensitive to the structure(s) of the ligand(s). For example, Liu et al. found that [Ni(COD)_2_]/dppf was not a competent catalyst for the cross-coupling of phenyl triflate and aniline but that a modified ligand (1,1′-bis(di(3,5-trifluoromethylphenyl)phosphino)ferrocene) enabled the reaction to achieve almost quantitative conversion.^6^ There are many examples of situations where the mechanisms of nickel-catalyzed reactions can also be very sensitive to the ligand structure. If the nickel-catalyzed Suzuki–Miyaura coupling of benzylic esters is carried out using tricyclohexylphosphine as the ligand, the stereochemistry at the benzylic position is retained; however, the use of SIMes produces the stereoinverted product via a different mechanistic pathway.^7^ The size of the NHC ligand in [Ni(NHC)_2_] complexes determines whether [Ni(Ar)X(NHC)_2_] or [NiX(NHC)_2_] products result from their reactions with aryl halides.^8,9^ The choice of ligand type—bisphosphine or bipyridine—is crucial in the trifluoromethylthiolation reactions of aryl halides.^10^ Changes in the phosphine ligand structure can influence oxidative addition selectivity in the reactions of esters^11^ or change oxidative addition chemoselectivity entirely.^12^

The reactions of alkyl halides with nickel(0) have received relatively little attention compared to reactions of aryl halides and other aryl electrophiles.^13−17^ Baird has studied the reactions of various alkyl halides with [Ni(PPh_3_)_4_] via variable temperature ^1^H and ^31^P NMR spectroscopic analyses of reactions in situ.^18^ The major products from the reactions of iodoalkanes are alkanes and alkenes, with a nickel hydride species obtained as a minor product; this suggests that formal oxidative addition was followed by β-hydride elimination. The alkanes were proposed to arise from the generation of alkyl radicals by halide abstraction, which then abstract the hydride ligand from a nickel hydride complex. The trends observed in the reactivity of alkyl halides followed the stability of the corresponding radicals, consistent with an operative radical mechanism.

**Scheme 1:**
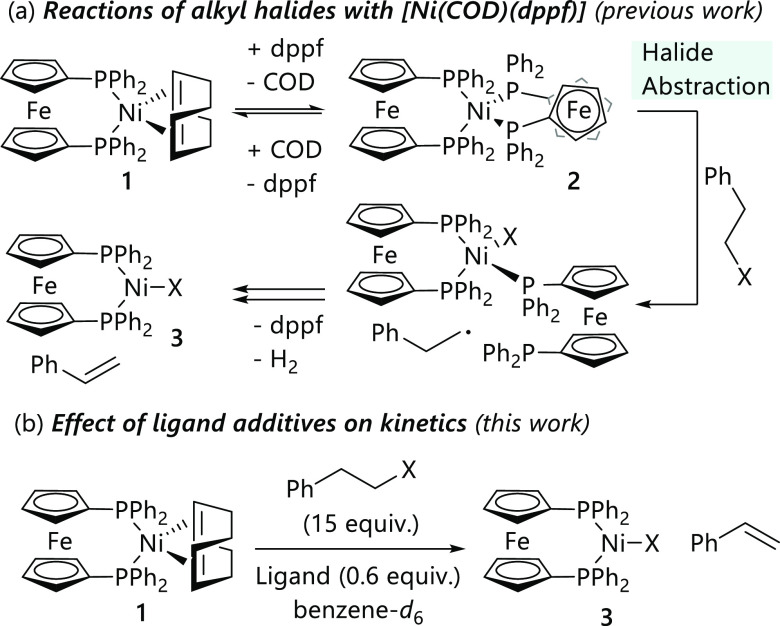
(a) Our Previous Study of the Reactions of [Ni(COD)(dppf)] with Alkyl Halides, and (b) This Work

We recently published a detailed study of the reactions of the model complex [Ni(COD)(dppf)]^19^ (**1**) with alkyl halides.^20^ The experimental and computational evidence that was gathered supported a mechanism in which [Ni(COD)(dppf)] was in equilibrium with [Ni(dppf)_2_] (**2**) (with the additional dppf being a trace impurity in **1**), and [Ni(κ^2^-dppf)(κ^1^-dppf)] performed a halide abstraction step to produce [Ni(X)(κ^2^-dppf)(κ^1^-dppf)] plus an alkyl radical (Scheme 1a); subsequent dppf dissociation and the recombination of the alkyl radical and nickel(I) complex yielded the formal oxidative addition product [Ni(X)(R)(dppf)], which underwent rapid β-hydride elimination. The final products were [Ni(X)(dppf)] (**3**) and alkene, with no alkane product observed.

The reaction of [Ni(COD)(dppf)] with alkyl halides relies upon the presence of additional dppf in order to form the three-coordinate species necessary for the halide abstraction step. However, the bidentate nature of dppf means that [Ni(dppf)_2_] is lower in energy than the desired [Ni(κ^2^-dppf)(κ^1^-dppf)] intermediate. Here, we have examined the use of a range of alternative monodentate ligands and their effects on the rate of stoichiometric and catalytic reactions of alkyl halides (Scheme 1b).

## Results and Discussion

### Kinetic Studies of Stoichiometric Reactions with Alternative Ligands

**Scheme 2:**
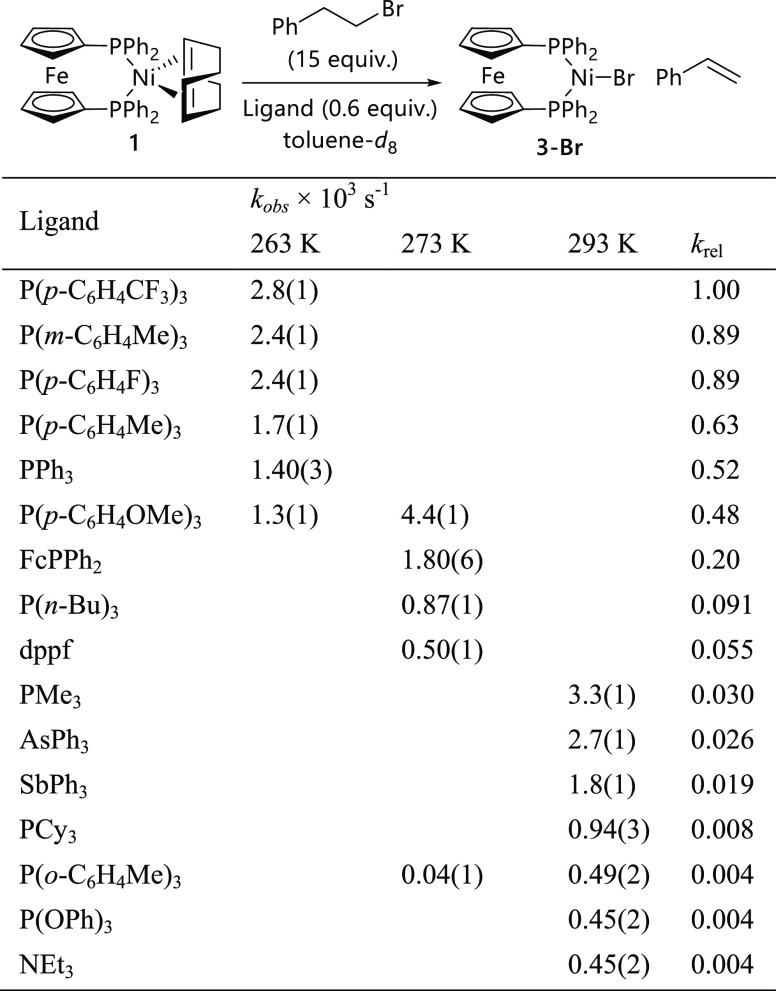
Kinetic Studies of the Reactions between [Ni(COD)(Dppf)] (**1**) (0.022 mol L^–**1**^) and (2-Bromoethyl)benzene (**4-Br**) (0.33 mol L^–**1**^) in Toluene-*d*_8_ in the Presence of Various Added Ligands (0.0132 mol L^–**1**^)

Our previous study^20^ established that the rate of reaction between [Ni(COD)(dppf)] (**1**) and alkyl halides was significantly increased by the addition of free dppf ligands, as this shifted the equilibrium between **1** and [Ni(dppf)_2_] toward the latter species. For this study, a selection of monodentate group 15 ligands were assembled with a diverse range of steric and electronic properties and where the Lewis basic atom was nitrogen, phosphorus, arsenic, or antimony. These were all used as additives in stoichiometric reactions between **1** and (2-bromoethyl)benzene (**4-Br**) which were monitored by ^31^P NMR spectroscopy. All experiments were pseudo-first-order in **1**, and the ^31^P NMR spectra confirmed that dppf remained bound to the nickel center throughout, with no free dppf ligand detected (δ_P_ = −17 ppm). Data were collected at one or two of three temperatures (263, 273, or 293 K) depending on how fast the reaction proceeded; the results for reactions with 15 monodentate ligands, along with previous data for the reaction with added dppf,^20^ are recorded in Scheme 2. Pseudo-first-order constants are listed in the order of largest to smallest. Data span a ca. 200-fold range of rate constants.

**Figure 1:**
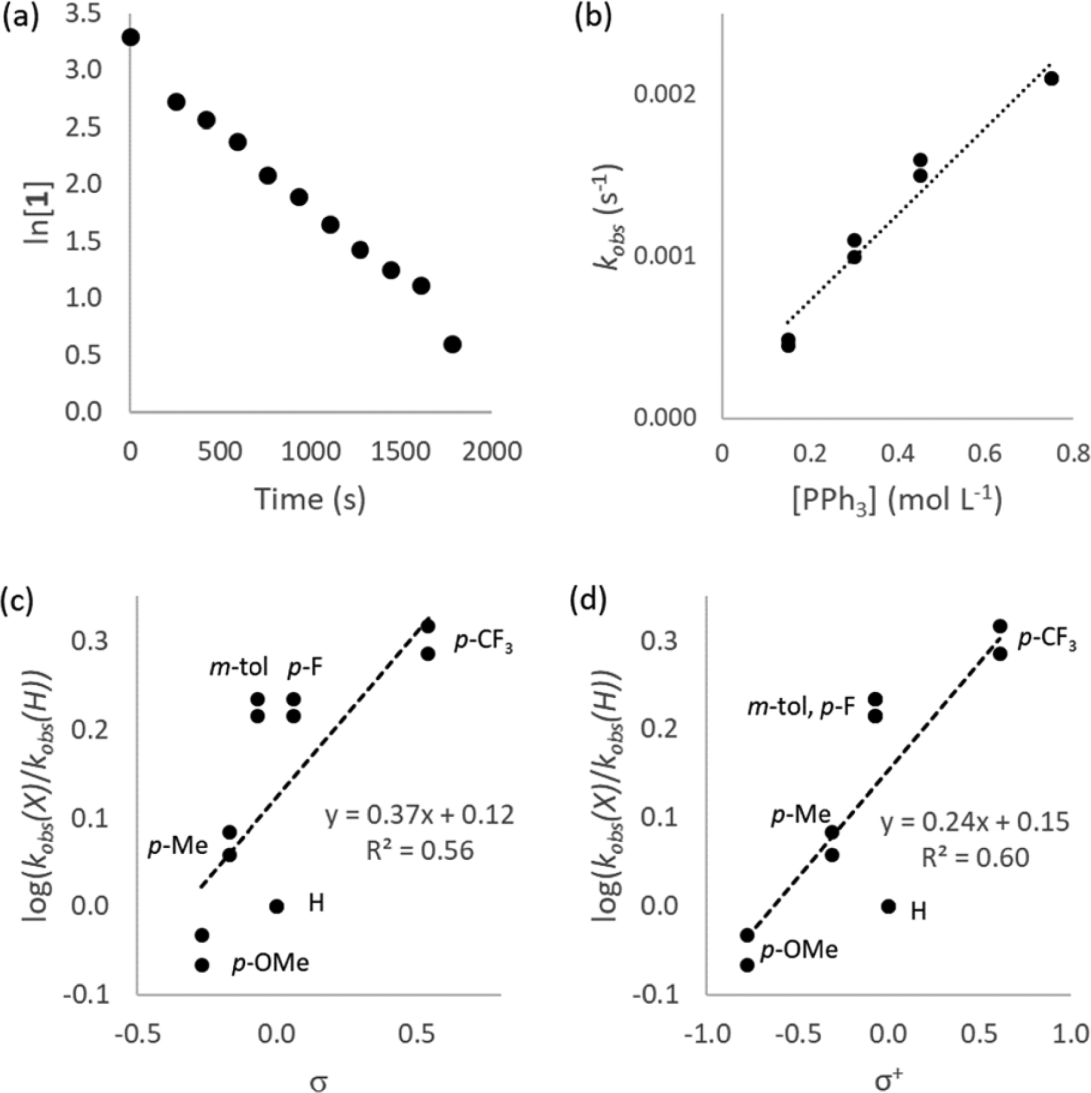
(a) Kinetic data for the reaction between [Ni(COD)(dppf)] (**1**) (0.022 mol L^–**1**^) and (2-bromoethyl)benzene (**4-Br**) (0.33 mol L^–**1**^) at 263 K in toluene-*d*_8_ in the presence of triphenylphosphine (0.0132 mol L^–**1**^). (b) Plot of *k_obs_* versus [PPh_3_]. (c) Hammett plot (using substituent constants σ) for the reactions in the presence of substituted triarylphosphines. (d) Hammett plot (using substituent constants σ^+^).

A series of reactions were carried out with different concentrations of triphenylphosphine, confirming that the reaction is first order in added triphenylphosphine (Figure 1a,b); our previous work noted that the reaction was first order in dppf when this was the added ligand.^20^

The use of diphenylphosphinoferrocene (FcPPh_2_) as an additive led to a higher rate of reaction than the corresponding experiment with dppf. This can be rationalized by considering the requirement for (bidentate) dppf to dissociate one phosphine atom from the nickel center to enable the reaction to occur; the binding of a second FcPPh_2_ ligand does not benefit from the chelate effect.

It is apparent that neither the steric nor the electronic properties of the ligand dominate the observed effects on reaction rates; The Tolman electronic parameter (TEP) and cone angle data are gathered for some of the ligands deployed in this study (Table 1).^21^ It was initially anticipated that electron-rich ligands would generate a more reactive nickel(0) complex and therefore accelerate halide abstraction. However, the use of tricyclohexylphosphine leads to a very slow reaction with no conversion after 45 min at 273 K (*k_rel_* = 0.008); reactions in the presence of trimethylphosphine (*k_rel_* = 0.030) or tri(*n*-butyl)phosphine (*k_rel_* = 0.091) are faster. The reaction with triphenylphosphine as an additive led to a reaction that was faster still (*k_rel_* = 0.52), despite being less electron-rich.

**Table 1:** Relative Rate Constants for Selected Reactions where the TEP and Cone Angle Are Known^21^ for the Corresponding Ligand

ligand	TEP (cm^–1^)	cone angle (°)	%*V*_bur_	*k*_rel_
P(*m*-C_6_H_4_Me)_3_	2067.2			0.89
P(*p*-C_6_H_4_F)_3_	2071.3		31.4	0.89
P(*p*-C_6_H_4_Me)_3_	2066.7	145	31.3	0.63
PPh_3_	2068.9	145	31.2	0.52
P(*p*-C_6_H_4_OMe)_3_	2066.7		31.3	0.48
P(*n*-Bu)_3_	2060.3	132		0.091
PMe_3_	2064.1	118	24.0	0.030
PCy_3_	2056.4	170	31.9	0.008
P(*o*-C_6_H_4_Me)_3_	2066.6	194		0.004
P(OPh)_3_	2085.3	128		0.004

A Hammett plot^22^ of log(*k*_obs(X)_/*k*_obs(H)_) versus σ for a set of five triarylphosphines gave a relatively shallow gradient of ρ = 0.37 (Figure 1c).^23^ A slightly better correlation with ρ = 0.24 is obtained using σ^+^ parameters for the *para*-substituted triarylphosphines (Figure 1d),^24^ but in both cases these show that the reaction is promoted by electron-poor triarylphosphines. The relatively simple reaction mechanism that we had initially anticipated—i.e., ligand binding, halide abstraction, and ligand dissociation—is too simple to explain the observed trends, and so, we turned to computational chemistry for additional insight.

### DFT Calculations of the Reaction Mechanism

Density functional theory (DFT) calculations carried out during our earlier study^20^ supported the proposal that the reaction occurs via formation of a three-coordinate nickel(0) complex, halide abstraction to form nickel(I) plus a radical, and recombination of these species to form a nickel(II) complex. The nickel(II) complex is then proposed to undergo β-hydride elimination followed by comproportionation to form [NiX(dppf)] (**3**), styrene, and hydrogen. For details of the level of theory used in this study please see the Experimental Section. Trimethylamine was used as a conformationally less complicated model for triethylamine; we have previously used trimethylphosphine as a model for triethylphosphine.^25^

**Scheme 3:**
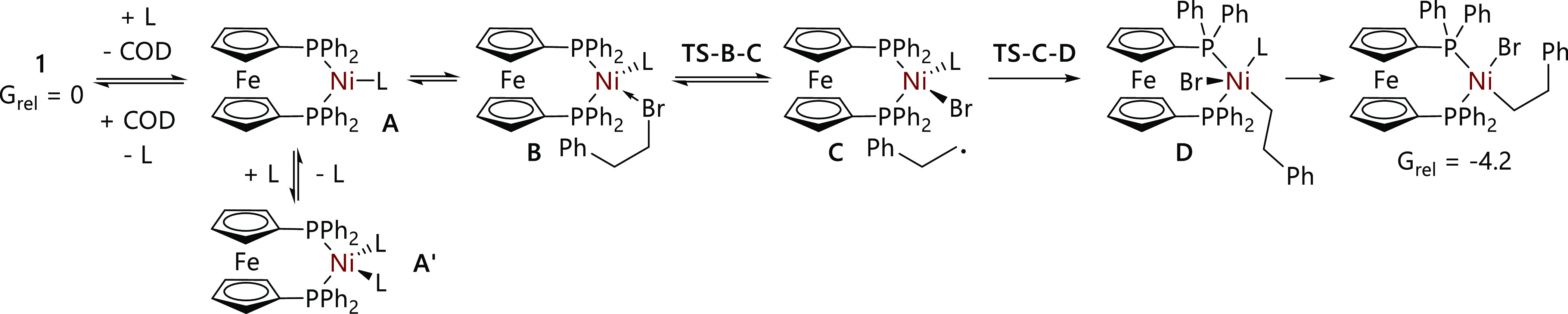
Mechanism for the Reactions of **1** Plus the Added Ligand (L) with (2-Bromoethyl)benzene **4-Br**

The reactions of [Ni(COD)(dppf)] (**1**) with (2-bromoethyl)benzene (**4-Br**) in the presence of added ligands were systematically studied. Scheme 3 outlines the mechanism, while Table 2 records the corresponding data and the overall barrier for the halide abstraction transition state versus the lowest energy preceding intermediate. For simplicity, the reactions with the corresponding alkyl chloride and alkyl iodide were not studied here.

**Table 2:** Free Energies for the Complexes Considered during This Study, Obtained at the M06/6–311 + G(d,p),LANL2DZ(d,p)[Sb], SMD(Benzene)//B3LYP-D3/6-31G(d),LANL2TZ(f)[Ni,Fe],LANL2DZ(d,p)[Br,As,Sb] Level of Theory, and Quoted Relative to [Ni(COD)(dppf)] (**1**)

ligand	*k*_rel_	*G*_rel_ (kcal/mol)								
		A	A′	B	TS-B-C	C	TS-C-D	D	Δ*G*^‡^ (HA)	Δ*G*^‡^ (recomb.)
P(*p*-C_6_H_4_CF_3_)_3_	1.00	0.0	4.1	4.3	23.8	20.5			23.8	
P(*p*-C_6_H_4_F)_3_	0.89	1.0	2.9	2.8	22.5	4.5			22.5	
P(*p*-C_6_H_4_Me)_3_	0.63	1.6	5.8	3.8	22.7	18.9			22.7	
PPh_3_	0.52	1.4	5.3	2.5	24.6	17.7	26.4	10.8	24.6	26.4
P(*p*-C_6_H_4_OMe)_3_	0.48	1.0	3.2	4.2	21.4	3.5			21.4	
FcPPh_2_	0.20	0.3	8.0	3.0	24.7	7.2			24.7	
dppf	0.055	–0.2^a^	b	2.9	24.6	10.1			26.1	
PMe_3_	0.030	0.0	–12.2	5.5	22.2	5.9	4.5	4.1	34.4	16.7
AsPh_3_	0.026	1.4	–5.3	12.1	22.0	9.4			27.3	
SbPh_3_	0.019	0.9	–14.6	9.3	19.0	11.2			33.6	
PCy_3_	0.008	–0.2	c	4.5	26.3	–0.2	31.7	14.6	26.3	31.9
P(OPh)_3_	0.004	–6.1	–21.6	–3.3	18.8	–13.1			40.4	
NMe_3_	0.004	14.3	c	21.1	32.8	15.1			32.8	

a [Ni(dppf)_2_] has *G*_rel_ = −1.5 kcal/mol.

b [Ni(dppf)(κ^1^-dppf)_2_] is unlikely to be competitive with [Ni(dppf)_2_]; note that [Ni(dppf)(FcPPh_2_)_2_] has *G*_rel_ = 8.0 kcal/mol.

c Attempts to locate structures for [Ni(dppf)(PCy_3_)_2_] and [Ni(dppf)(NMe_3_)_2_] led to spontaneous dissociation of one of the ligands during geometry optimization.

In most cases, [Ni(dppf)(L)] had *G*_rel_ = −0.6 to 1.6 kcal/mol, with the exceptions of trimethylamine (*G*_rel_ = 14.3 kcal/mol) and triphenylphosphite (*G*_rel_ = −6.1 kcal/mol). The strong binding of the π-accepting phosphite to the dppf-nickel(0) fragment is not unexpected, given the important role of π-backbonding in the coordination chemistry of organometallic complexes of nickel(0).^26−28^ The steric profile of each coordinated ligand in a selection of the corresponding [Ni(dppf)(L)] complexes was evaluated using the percent buried volume (%*V*_bur_) metric (see the Supporting Information for the full data set).^29−31^ This metric has been widely applied across organometallic chemistry and catalysis.^32,33^ The %*V*_bur_ value did not vary as much as was anticipated. For example, tricyclohexylphosphine and triphenylphosphine have %*V*_bur_ that vary by less than one unit (31.9 and 31.2, respectively), despite their vastly different cone angles (170° and 145°, respectively). Trimethylphosphine (24.0), triphenylarsine (22.7), and triphenylstibene (27.7) have a lower %*V*_bur_ than triarylphosphines (ca. 31) in this environment.

It is possible for two monodentate ligands to coordinate the [Ni(dppf)] fragment in most cases; [Ni(dppf)(PMe_3_)_2_]^34^ and [Ni(dppf)(P(OPh)_3_)_2_]^35^ are known species that have been fully characterized using methods including single crystal X-ray diffraction. The possible formation of these species was also investigated computationally. In the case of trimethylamine and tricyclohexylphosphine, geometry optimization led to the spontaneous decoordination of the second ligand. In all other cases, [Ni(dppf)(L)_2_] complexes could be optimized as minima on the free energy surface. For triphenylarsine, triphenylstibene, trimethylphosphine, and triphenylphosphite the binding of a second ligand is very favorable, and so this increases the barrier to halide abstraction by [Ni(dppf)(L)]; this explains the rather poor performance of these four ligands in the kinetic experiments. The binding of a second diphenylphosphinoferrocene or triarylphosphine ligand is endergonic by a few kcal/mol.

The next step is the formation of [Ni(BrCH_2_CH_2_Ph) (dppf)(L)], although in many cases the steric environment around nickel precludes short Ni ... Br distances. These are typically slightly higher in energy than [Ni(dppf)(L)], presumably due to the entropic cost of bringing two molecules together. Halide abstraction takes place subsequently, and forms [NiBr(dppf)(L)] plus an alkyl radical. Our initial treatment of the data assumed facile ligand dissociation and radical recombination to form [NiBr(CH_2_CH_2_Ph)(dppf)] (**5**) (*G*_rel_ = −4.2 kcal/mol) which transpired to be an oversimplification of the reaction mechanism;^20^ the events after halide abstraction but before the formation of **5** will be discussed subsequently.

An initial analysis of the data revealed limited agreement between experimentally determined rate constants and computationally determined halide abstraction barriers. FcPPh_2_ is a more effective ligand than dppf, and this is reflected in the 1.4 kcal/mol decrease in Δ*G*^‡^. However, the DFT data for triarylphosphine ligands are at odds with the experimental observations and instead suggest that the reactions with more electron-rich ligands should proceed more quickly.

We next considered the possibility that the halide abstraction is in fact reversible and that a subsequent step in the mechanism might be rate-determining in some or all cases. Experimental evidence suggests that the alkyl radical exists for long enough to undergo unimolecular rearrangement reactions, but the lack of any corresponding alkane or dimerized product suggests that it is captured by the nickel complex relatively quickly.^20^ However, the radical might be captured by the formation of a nickel(II) complex (formation of a C–Ni bond) or by the abstraction of the halide from the nickel center (C–X reformation), especially within the relatively crowded environment of the nickel center.

Further calculations identified transition states for the combination of the alkyl radical with [NiBr(dppf)(L)], where L is trimethylphosphine, tricyclohexylphosphine, or triphenylphosphine. In the case of trimethylphosphine, two transition states were characterized: one with approximately trigonal bipyramidal geometry and one (of lower energy) with distorted square-based pyramidal geometry. For tricyclohexylphosphine and triphenylphosphine pathways only the latter geometry of the transition state was located; attempts to locate trigonal bipyramidal transition states were unsuccessful. Geometry optimizations of structures along the reaction coordinate confirmed that the transition states linked the nickel(I) complex and a square-based pyramidal nickel(II) species.

The barrier to recombination varies considerably depending on the identity of the ligand. For tricyclohexylphosphine, the radical capture transition state is significantly higher in energy than the halide abstraction transition state (31.7 versus 26.3 kcal/mol), which explains the poor performance of this ligand in the stoichiometric reactions. In contrast, the recombination of the radical with the trimethylphosphine complex presents no significant barrier.

**Figure 2:**
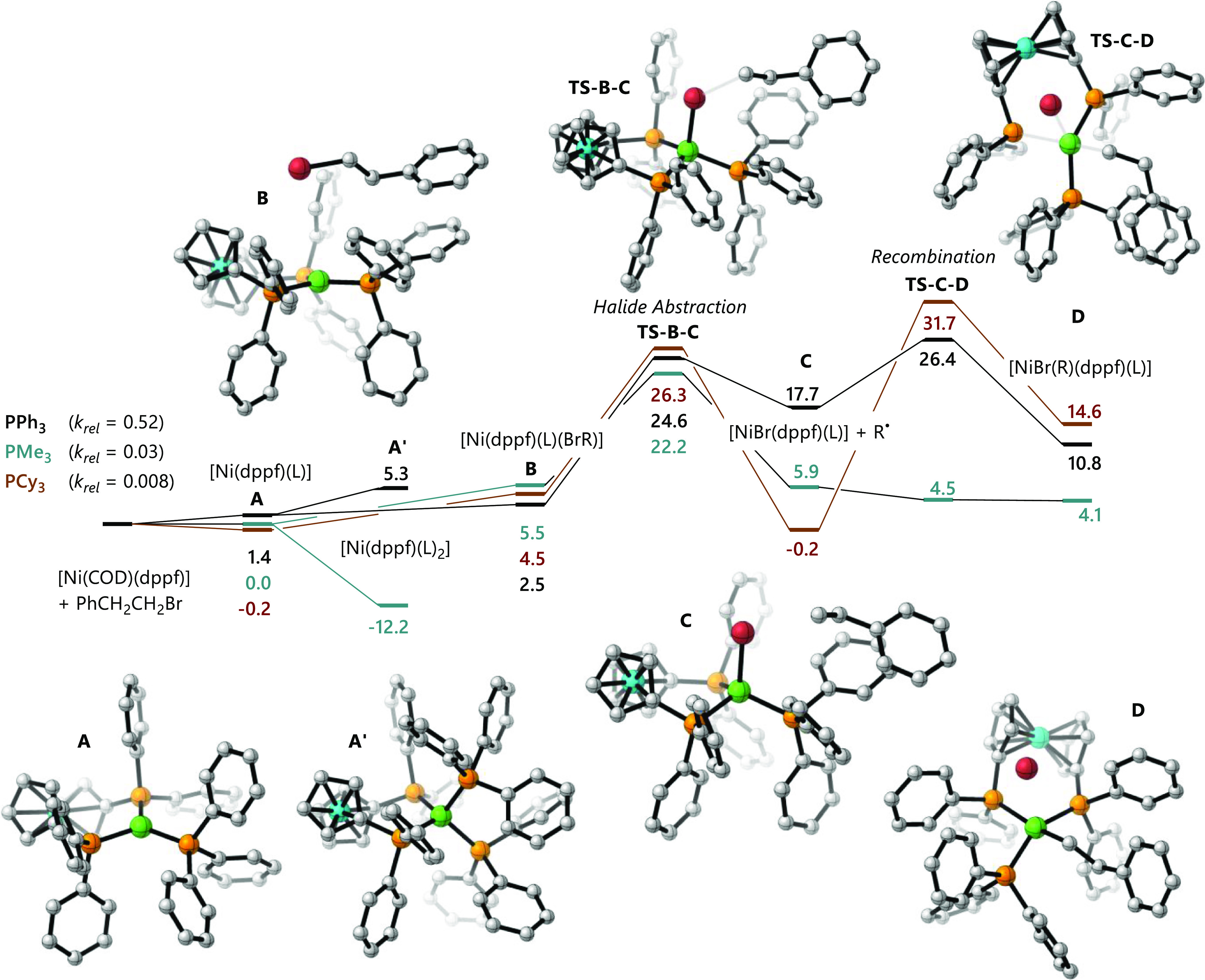
Free energy profiles for the reactions of [Ni(COD)(dppf)] (**1**) with (2-bromoethyl)benzene (**4-Br**) in the presence of triphenylphosphine (black), trimethylphosphine (teal), or tricyclohexylphosphine (red). Images represent DFT-derived structures of the relevant intermediates with triphenylphosphine as the added ligand (Ni green, Fe blue, P orange, Br red, and C gray) with H atoms omitted for clarity.

Figure 2 displays the free energy profiles for the reactions where trimethylphosphine, tricyclohexylphosphine, and triphenylphosphine are used as additives; these illustrate the three types of behavior that are observed in these reactions. These are discussed in turn.

In the case of trimethylphosphine, the formation of [Ni(dppf)(PMe_3_)_2_] inhibits the reaction because one of the trimethylphosphine ligands must dissociate before halide abstraction can occur, and this carries a significant energetic penalty. Similar behavior is observed for triphenylphosphite, triphenylarsine, and triphenylstibene.

The reaction in the presence of tricyclohexylphosphine suffers from a large barrier to radical recombination with the nickel(I) intermediate. A structure for [Ni(dppf)(PCy_3_)_2_] could not be obtained because one of the tricyclohexylphosphine ligands dissociated during geometry optimization, and so, the barrier to halide abstraction is reasonable. However, the radical formed during halide abstraction (**D**) faces a smaller barrier to re-form the C–Br bond (26.5 kcal/mol) than to form a C–Ni bond (31.9 kcal/mol), and so the halide abstraction is reversible.

The reaction in which triphenylphosphine is present faces neither of these issues. The coordination of a second phosphine (Δ*G* = 3.9 kcal/mol) is less favorable than coordination of the substrate (Δ*G* = 1.1 kcal/mol) and the transition states for C–Br formation and C–Ni formation are close in energy (*G*_rel_ = 24.6 and 26.4 kcal/mol), respectively.

The “ideal” added ligand for this process is therefore a ligand that coordinates only once but is not sufficiently bulky to interfere with the recombination of the radical with nickel(I).

### Relevance to Catalysis

**Scheme 4:**
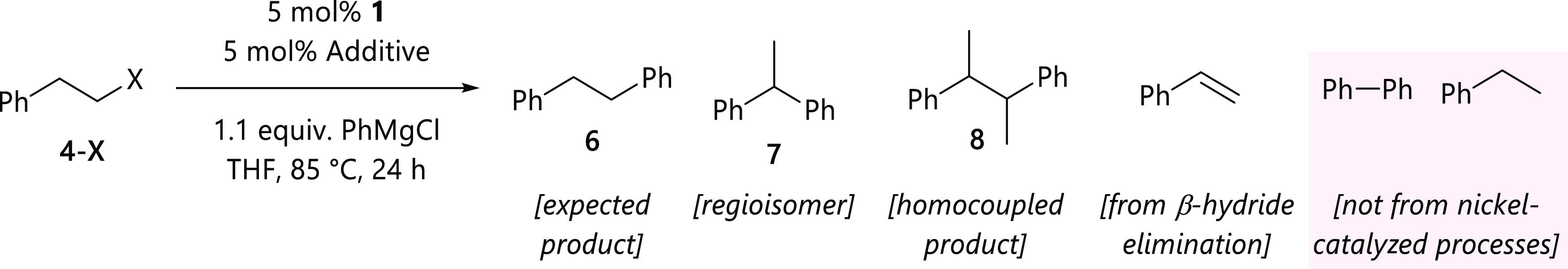
Model Kumada–Tamao–Corriu Reactions

We sought to link our new understanding of the effects of ligands on the halide abstraction step to the outcomes of catalytic reactions of importance to synthetic chemistry. A series of prototypical Kumada–Tamao–Corriu cross-coupling reactions were carried out using (2-haloethyl)benzene substrates (**4-Cl**, **4-Br**, **4-I**) to understand the effects of additives on catalytic reactions (Scheme 4). All reactions were catalyzed by 5 mol % [Ni(COD)(dppf)] in the presence of 5 mol % of an additional ligand; the same conditions were used in our previous study.^20^

These reactions, and reactions closely analogous to these, have previously been carried out using other nickel catalysts and typically at temperatures around room temperature. Catalyst systems include 10 mol % Cp*CH_2_PPh_2_/5 mol % NiCl_2_,^36^ 5 mol % of a nickel pincer complex,^37^ 2.5 mol % of a dinickel(II)/tridenate nitrogen ligand complex,^38^ and 2 mol % of a diphosphinodithio complex of nickel.^39^ The topics of nickel-catalyzed alkyl halide cross-coupling reactions^40,41^ and nickel-catalyzed Kumada–Tamao–Corriu reactions have been reviewed.^42^

**Figure 3:**
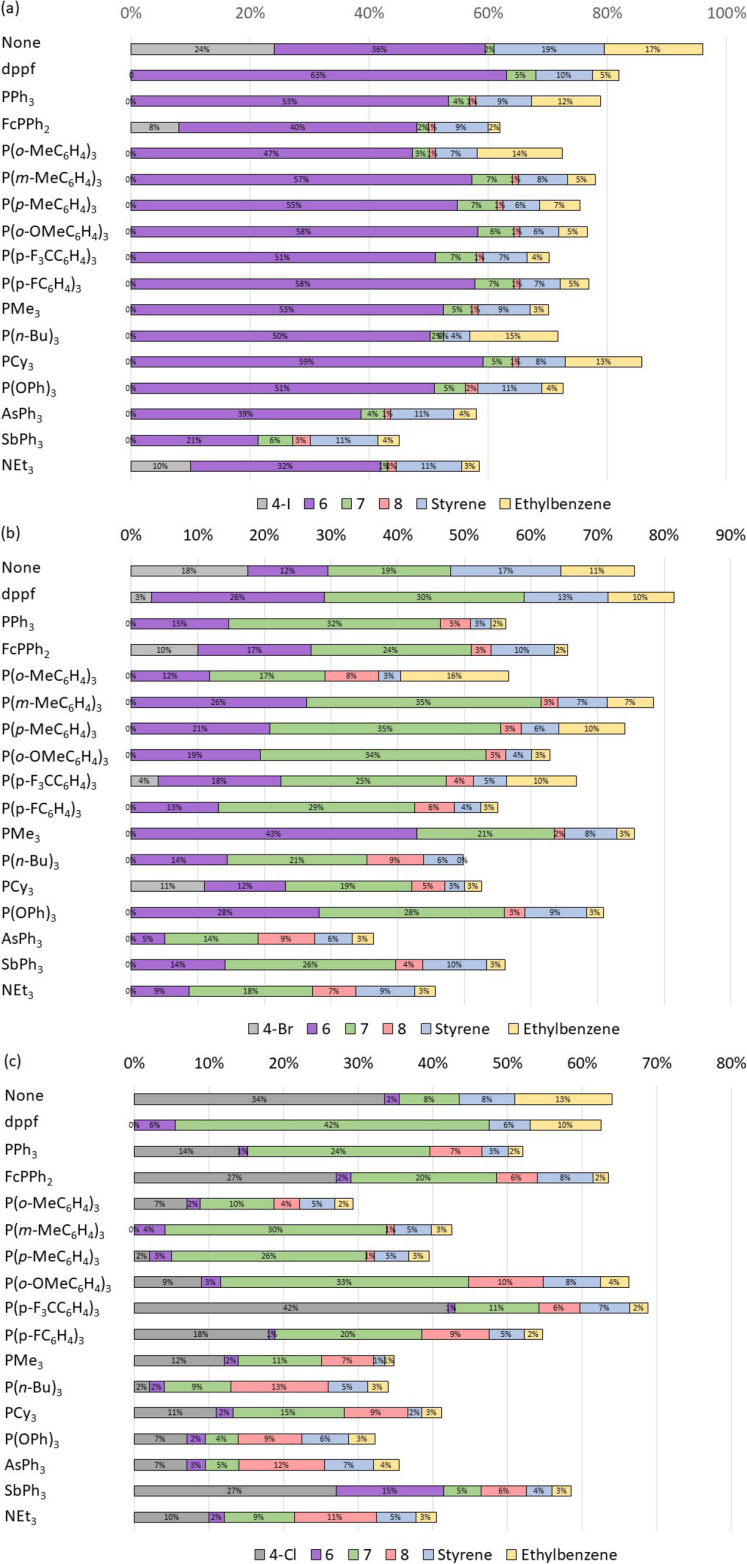
Product distributions in model Kumada–Tamao–Corriu cross-coupling reactions using different added ligands. Reactions were conducted with three substrates: (a) (2-iodoethyl)benzene, (b) (2-bromoethyl)benzene, and (c) (2-chloroethyl)benzene.

The reactions undertaken for this study, like those disclosed previously,^20^ produced the expected 1,2-diphenylethane product (**6**), the 1,1-diphenylethane regioisomer (**7**), styrene, ethylbenzene, and biphenyl (Figure 3). We have shown previously, through the use of control reactions, that ethylbenzene and biphenyl do not arise from nickel-catalyzed reactions.^20^ The regioisomer is likely formed from β-hydride elimination followed by migratory insertion to generate [NiX(CH(Me)Ph)(dppf)].^20^ The alkyl halide homocoupling product 2,3-diphenylbutane (**8**) was observed in some reactions, particularly when **4-Cl** was used as the substrate. All reaction outcomes were quantified using gas chromatography-flame ionization detection (GC-FID) analysis with an internal standard; the gas chromatography-flame ionization detector was calibrated using authentic pure samples of each analyte. These model reactions proceed poorly in the absence of an added ligand. The reactions of **4-I** are relatively insensitive to the choice of the added ligand, although triphenylstibene and triethylamine perform quite poorly. It is likely that the relatively weak carbon–iodine bond strength means that the halide abstraction step is unlikely to be rate-determining in the reactions of alkyl iodides. These reactions significantly favor the expected (linear) product (**6**) over the branched side-product (**7**). The reactions of **4-Br** show more diverse outcomes and produce mixtures of **6** and **7**. Here, the most effective ligands appear to be trimethylphosphine, tri(*meta*-tolyl)phosphine, and triphenylphosphite. The reactions of **4-Cl** are evidently more challenging, and most reactions had rather poor mass balance and/or did not achieve complete substrate conversion. Once again, tri(*meta*-tolyl)phosphine appears to be the best ligand in terms of enabling complete conversion of the alkyl halide substrate, although the major product is regioisomer **7**.

Despite the 200-fold spread of reaction rates in the stoichiometric halide abstraction reactions, the outcomes of catalysis are generally rather less variable. This suggests that the halide abstraction step is not rate-determining within the overall catalytic cycle and/or that the more forcing conditions used for catalysis mitigate the energetic barrier involved for some ligands. Tri(*meta*-tolyl)phosphine emerges as a ligand that is a good choice for the reactions of any of the three of these substrates. It must be noted that a full optimization of this reaction has not been carried out, but the choice of the added ligand represents an important factor that should be considered in these reactions. This added ligand can of course affect steps other than halide abstraction, but an examination of the full catalytic cycle for this reaction is beyond the scope of the present study.

## Conclusions

This study has established that a range of different added ligands increase the rate of the reaction between [Ni(COD)(dppf)] (**1**) and a model alkyl bromide (**4-Br**), with a ca. 200-fold range of rate constants. This is consistent with our current mechanistic model which requires a three-coordinate nickel(0) complex that can abstract the halide atom from the alkyl halide substrate. DFT studies have provided further insight into the reaction, identifying that the rate-determining step in the stoichiometric reactions between **1** and **4-Br** can be either the halide abstraction step or the recombination of the alkyl radical with the nickel(I) complex formed during halide abstraction.

Studies of a prototypical Kumada–Tamao–Corriu reaction have established that the choice of ligand has relatively little effect for the reactions of **4-I** but that the reactions in the absence of an added ligand give poor outcomes. The outcomes of the reactions of **4-Br** and **4-Cl** show a more complicated dependence on the structure of the added ligand, but this certainly presents a useful vector for the optimization of these types of cross-coupling reactions.

Further studies of the complex reactions between nickel(0) and alkyl halides and of the related catalytic cross-coupling reactions are currently underway within our laboratories.

The raw data underpinning the experimental parts of this study can be downloaded from the University of Strathclyde Knowledgebase at http://dx.doi.org/10.15129/10523ca4-2c98-4d77-8575-06d97ba66621. Computational chemistry data underpinning this study can be accessed via the ioChem-BD data repository^43^ at http://dx.doi.org/10.19061/iochem-bd-1-208.

## Experimental Section

### Materials

Anhydrous toluene, THF, and hexane were obtained from an Inert Technologies PureSolv apparatus (<10 ppm water by Karl-Fischer titration). Any manipulations of air-sensitive nickel complexes were carried out under argon using Schlenk techniques or in a glovebox. Substrates used for kinetic experiments and cross-coupling reactions were obtained from commercial sources and used as supplied. The synthesis of FcPPh_2_ and some of the reaction side products are detailed below; characterization data for the remaining products can be found in our previous paper.^20^ [Ni(COD)_2_] (96% purity) was purchased from Alfa Aesar and stored at −35 °C in a glovebox freezer. 1,1′-Bis(diphenylphosphino)ferrocene was purchased from Fluorochem and stored in a glovebox. [Ni(COD)(dppf)] was prepared according to the literature method.^19^ Deuterated solvents were obtained from commercial sources and dried overnight on 4 Å molecular sieves before use.

### Analysis

NMR spectroscopy was performed using Bruker AV3–400 (liquid nitrogen cryoprobe), Bruker AV3–400 Nano (BBFO-z-ATMA probe), or Bruker AVII-600 (BBO-z-ATMA) instruments. All kinetic experiments were performed using the latter instrument. ^1^H NMR spectra are referenced to residual solvent signals, ^13^C{^1^H} NMR spectra are referenced to the deuterated solvent signal, and ^31^P{^1^H} NMR spectra are externally referenced.^44^ Chemical shifts are given in ppm and coupling constants in Hertz. Gas chromatography–mass spectrometry (GC–MS) analyses were carried out using an Agilent 7890A gas chromatograph fitted with a RESTEK-RXi-5Sil column (30 m × 0.32 mm I.D. × 0.25 μm) connected to an Agilent 5975C MSD running in an EI mode. GC-FID analyses were carried out using an Agilent 7890A gas chromatograph fitted with an Agilent HP5 column (30 m × 0.25 mm I.D. × 0.25 μm).

### 1,4-Diphenylbutane

1,4-Diphenyl-1,3-butadiene (1 g, 4.8 mmol) was added to a flask with Pd/C (0.1 g, 10 wt %), which was then sealed with a septum and evacuated and backfilled with nitrogen. Propan-2-ol (20 mL) was added and the flask was evacuated and backfilled again. A balloon of hydrogen was attached to the flask via a needle. The reaction was stirred at room temperature for 24 h. The balloon was removed, the Pd/C was filtered off, and the solvent was removed under reduced pressure to give 1,3-diphenylbutane as a white solid (0.7 g, 73%). ^1^H NMR (400 MHz, CDCl_3_): δ 1.71 (d*t*, 4H,^3^*J*_H–H_ = 7.0, ArC**H**_2_), 2.67 (*t*, 4H,^3^*J*_H–H_ = 7.1, ArCH_2_C**H**_2_), 7.19–7.22 (*m*, 6H, aryl C–**H**), 7.28–7.32 (*m*, 4H, aryl C–**H**). ^13^C{^1^H} NMR (101 MHz, CDCl_3_): δ 30.6, 35.3, 125.1, 127.8, 127.9, 142.2. GC–MS (C_16_H_18_) *m*/*z*: 210.2. NMR data are consistent with the literature.^45^

### 2,3-Diphenylbutane

Benzil (2 g, 9.5 mmol) was dissolved in anhydrous THF (20 mL) under a nitrogen atmosphere. MeMgCl (12.6 mL, 3 M in THF, 37.8 mmol) was added dropwise and the mixture was stirred at room temperature for 16 h. The reaction mixture was quenched (HCl, 1 M, 100 mL) and extracted with DCM (3 × 20 mL). The organic layers were combined, dried over MgSO_4_, filtered, and concentrated to give 2,3-diphenylbutane-2,3-diol which was used in the next step without purification. Following a literature preparation,^46^ 2,3-diphenylbutane-2,3-diol (1.5 g) was dissolved in hexamethylphosphoramide (20 mL) and stirred at room temperature for 2 h. The mixture was then heated to reflux for 90 min. After cooling, Et_2_O (25 mL) was added and the reaction mixture was washed with water (100 mL). The aqueous layer was extracted with Et_2_O (15 mL). The combined organic layers were then washed with water (2 × 50 mL) and brine (25 mL), dried over MgSO_4_, filtered, and concentrated to give brown/red oil. Column chromatography on silica gel (petroleum ether) gave 2,3-diphenylbuta-1,3-diene as a white solid. 2,3-Diphenylbuta-1,3-diene (150 mg) was added to a RBF with Pd/C (15 mg, 10 wt %), which was then sealed with a septum and evacuated and backfilled with nitrogen. Propan-2-ol (20 mL) was added and the flask was evacuated and backfilled again. A balloon of hydrogen was attached to the flask via a needle. The reaction was then stirred at room temperature for 24 h. The balloon was removed, the Pd/C was filtered off and the solvent was removed under reduced pressure to give 2,3-diphenylbutane (95 mg, 5% over three steps) as a white solid. ^1^H NMR (400 MHz, CDCl_3_): δ 1.05 (dd, 6H,^3^*J*_H–H_ = 2.2, 6.7), 1.31 (dd, 4H,^3^*J*_H–H_ = 1.9, 6.7), 2.80–2.84 (*m*, 2H), 2.93–3.00 (*m*, 1.3H), 7.02–7.04 (*m*, 2.5H), 7.09–7.14 (*m*, 1.3H), 7.17–7.26 (*m*, 8.2H), 7.32–7.36 (*m*, 3.8H). ^13^C{^1^H} NMR (101 MHz, CDCl_3_): δ 17.4, 20.5, 45.9, 46.8, 125.2, 125.5, 127.1, 127.2, 127.3, 127.8, 145.9. GC–MS (C_16_H_18_) m/z: 210.2. NMR data are consistent with the literature.^47^

### Diphenylphosphinoferrocene

This was prepared according to a literature procedure.^48^ Ferrocene (1.9 g, 0.1 mmol) and aluminum chloride (1.3 g, 0.1 mmol) were dissolved in hexane (20 mL). Chlorodiphenylphosphine was added and the solution was heated at reflux for 16 h. The hexane was decanted and solids were extracted with fresh hexane (50 mL). This was repeated with water (50 mL). The hexane and water were discarded, and the remaining solids were extracted with hot toluene (50 mL). The toluene was dried over MgSO_4_, filtered, and concentrated to dryness. The residue was extracted with hexane (100 mL) and this was concentrated to yield the product as yellow powder (92 mg, 3%). ^1^H NMR (400 MHz, CDCl_3_): δ 4.12 (q, 4H,^3^*J*_H–H_ = 1.8 Hz), 4.39 (*t*, 4H,^3^*J*_H–H_ = 1.8 Hz), 7.33–7.41 (*m*, 20H). ^13^C{^1^H} NMR (101 MHz, CDCl_3_): δ 68.6, 70.2 (d, *J*_P–C_ = 3.9), 72.4 (d, *J*_P–C_ = 14.9), 127.6 (d, *J*_P–C_ = 14.9), 127.9, 132.9, 133.1, 138.6 (d, *J*_P–C_ = 9.6). ^31^P{^1^H} NMR (162 MHz, CDCl_3_): δ −17.0.

### Kinetic Experiments

Kinetic data were obtained using the same methodology that has been deployed previously, by monitoring the decay of the concentration of [Ni(COD)(dppf)] over time by ^31^P or ^31^P{^1^H} NMR spectroscopy.^13,20,28^ For a typical experiment, in an argon-filled glovebox, a septum-fitted NMR tube was charged with a benzene-*d*_6_ or toluene-*d*_8_ solution containing [Ni(COD)(dppf)] (8 mg, 0.011 mmol) and any solid additives. Any liquid additives—or stock solutions of additives—were then added. The total volume of the reaction was 0.5 mL This sample was used to tune, match, lock, and shim the spectrometer. The alkyl halide was then added via a syringe through the septum to start the reaction, and ^31^P{^1^H} NMR spectra were acquired at intervals until ca. 87% conversion (16 scans, 2 s between scans). All experiments were performed in duplicate. In all reactions, pseudo-first-order rate constants (*k*_obs_) were obtained from a linear plot of the natural log of the integral of the signal for [Ni(COD)(dppf)] versus time. The rate constants from each experiment are tabulated in the Supporting Information.

### Cross-Coupling Reactions

In an argon-filled glovebox, [Ni(COD)(dppf)] (0.0125 mmol, 5 mol %) and any solid additives were added to a microwave vial equipped with a stirrer bar. The vial was sealed with a septum-fitted cap and removed from the glovebox. On a Schlenk line, anhydrous THF (1 mL) was added followed by phenylmagnesium chloride (0.28 mmol, 1.1 equiv). The vial was then heated (with rapid stirring) to 85 °C. When at the desired temperature, any liquid additives and the aryl halide (0.25 mmol) were added via a microsyringe. The reactions were stirred at 85 °C for 24 h, then cooled to room temperature, and pierced with a needle. Each vial was opened, an accurately known amount of *n*-tetradecane was added, and a sample was taken for GC-FID analysis. All reactions were performed in duplicate; average conversions are reported in the paper, and the result of each individual cross-coupling reaction is tabulated in the Supporting Information. The gas chromatography-flame ionization detector was calibrated for each substrate and product using authentic samples of each compound.

### Computational Methodology

DFT calculations were carried out using Gaussian16 Rev. A.03.^49^ DLPNO-CCSD(T) calculations^50−52^ were carried out using Orca 4.2.1.^53,54^ Geometry optimizations were carried out without symmetry constraints using the B3LYP functional^55−58^ with the Grimme D3 empirical dispersion correction.^59^ The LANL2TZ(f) pseudopotential/ECP was used for nickel and iron, while LANL2DZ(d,p) was used for bromine, arsenic, and antimony.^60−62^ The 6-31G(d) basis set was used for all other atoms. Frequency calculations verified the nature of stationary points. Transition states were checked using IRC calculations or by optimizing structures along the reaction coordinate. The energies of all structures were refined using single point calculations with the M06 functional,^63^ the LANL2DZ(d,p) pseudopotential/ECP on antimony, and the 6-311 + G(d,p) basis set on all other atoms. Solvation was included for the single point calculations using the SMD model (in benzene).^64^ This level of theory was decided upon by benchmarking calculations for the formation of [Ni(dppf)_2_] plus COD from [Ni(COD)(dppf)] plus dppf: Δ*G* (experiment) = −1.2 kcal/mol; Δ*G* (DLPNO-CCSD(T)/cc-pVTZ) = −1.6 kcal/mol; Δ*G* (M06/6-311 + *G*(d,p) = −1.5 kcal/mol (see the Supporting Information for full details). A correction of +1.89 kcal/mol was applied to the free energy of each species to consider a 1 mol/L reference state for the calculations rather than the ideal gas concentration.^65^ Yamaguchi’s approach was used to correct the electronic energies of open shell singlets for triplet contamination.^66^ The images of DFT-derived structures in Figure 2 were prepared using CYLView 2.0.^67^

## Supporting Information Available

Kinetic data, computational data (methods and energies), and data regarding the outcomes of catalytic reactions (PDF)
Coordinates of the structures modeled using DFT calculations (XYZ)

## Supplementary Material

om1c00280_si_001.pdf

om1c00280_si_002.xyz

## Abbreviations

DPPF: 1,1′-bis(diphenylphosphino)ferrocene;
SIMes: 1,3-bis(2,4,6-trimethylphenyl)-4,5-dihydroimidazol-2-ylidene
NHC: *N*-heterocyclic carbene
