# Supplementary material for: The Effect of Added Ligands on the Reactions of [Ni(COD)(dppf)] with Alkyl Halides: Halide Abstraction May Be Reversible
Source: Organometallics. 2021 Jun 16;40(12):1997–2007. doi: 10.1021/acs.organomet.1c00280 (PMC8288641; doi:10.1021/acs.organomet.1c00280)
Supplement: Supplementary file 1 — om1c00280_si_001.pdf [file om1c00280_si_001.pdf]

SUPPORTING INFORMATION FOR:

The Effect of Added Ligands on the Reaction of [Ni(COD)(dppf)] with Alkyl Halides: Halide Abstraction may be Reversible

Megan. E. Greaves,[a,b] Thomas O. Ronson,[b] Feliu Maseras,[c] and David J. Nelson.[a]

[a] WestCHEM Department of Pure and Applied Chemistry, University of Strathclyde, 295 Cathedral Street, Glasgow, G1 1XL, Scotland. Email: david.nelson@strath.ac.uk

[b] Chemical Development, Pharmaceutical Technology and Development, Operations, AstraZeneca, Macclesfield, SK10 2NA, UK.

[c] Institute of Chemical Research of Catalonia, Barcelona Institute of Science and Technology, Av. Països Catalans 16, 43007 Tarragona, Spain.

Contents

1. Kinetic Experiments 2
 1.1. Rates of Oxidative Addition 2
2. Cross-Coupling Reactions 4
 2.1. Results from Cross-Coupling Reactions 4
 2.2. GC-FID Calibrations 8
3. NMR Spectra 9
4. Kinetic Plots 13
 4.1. Data for Table S1 13
 6.2. Data for Table S2 18
5. Computational Chemistry 20
 5.1 Methodology 20
 5.2 $\%V_{bur}$ Calculations for L in [Ni(dppf)(L)] 20
 5.3 Benchmarking Studies 21
 5.4 Table of Energies 22
 5.5 Coordinates 25
6. References 93

1. Kinetic Experiments

1.1. Rates of Oxidative Addition

Table S1. Pseudo-first order rate constants obtained from plotting the natural log of the concentration of Ni^0 versus time (first-order dependence). All reactions carried out with Ni(COD)(dppf) (**1**) (22 mmol L^{-1}) 2-bromoethylbenzene (0.3 mol L^{-1}) as substrate and 13.2 mmol L^{-1} of each additive in 0.5 mL toluene-d_8.

**Entry**	**Additive**	**T (K)**	**k_{obs} (s^{-1})**
1a	P(*p*-$C_6H_4CF_3$)$_3$	263	2.9×10^{-3}
1b	P(*p*-$C_6H_4CF_3$)$_3$	263	2.7×10^{-3}
2a	P(*m*-C_6H_4Me)$_3$	263	2.4×10^{-3}
2b	P(*m*-C_6H_4Me)$_3$	263	2.3×10^{-3}
3a	P(*p*-C_6H_4F)$_3$	263	2.3×10^{-3}
3b	P(*p*-C_6H_4F)$_3$	263	2.4×10^{-3}
4a	P(*p*-C_6H_4Me)$_3$	263	1.6×10^{-3}
4b	P(*p*-C_6H_4Me)$_3$	263	1.7×10^{-3}
5a	PPh_3	263	1.38×10^{-3}
5b	PPh_3	263	1.43×10^{-3}
6a	P(*p*-C_6H_4OMe)$_3$	263	1.2×10^{-3}
6b	P(*p*-C_6H_4OMe)$_3$	263	1.3×10^{-3}
7a	P(*p*-C_6H_4OMe)$_3$	273	4.3×10^{-3}
7b	P(*p*-C_6H_4OMe)$_3$	273	4.4×10^{-3}
8a	P(*n*-Bu)$_3$	273	8.3×10^{-4}
8b	P(*n*-Bu)$_3$	273	9.1×10^{-4}
9a	(Diphenylphosphinyl)ferrocene	273	1.84×10^{-3}
9b	(Diphenylphosphinyl)ferrocene	273	1.75×10^{-3}
10a	dppf	273	4.9×10^{-4}
10b	dppf	273	5.0×10^{-4}
11a	PMe_3	293	3.2×10^{-3}
11b	PMe_3	293	3.3×10^{-3}
12a	$AsPh_3$	293	2.7×10^{-3}
12b	$AsPh_3$	293	2.6×10^{-3}
13a	$SbPh_3$	293	1.7×10^{-3}
13b	$SbPh_3$	293	1.8×10^{-3}
14a	2,2′-Bipyridine	293	1.4×10^{-3}
14b	2,2′-Bipyridine	293	1.7×10^{-3}
15a	PCy_3	293	9.6×10^{-4}
15b	PCy_3	293	9.1×10^{-4}
16a	Pyridine	293	7.3×10^{-4}
16b	Pyridine	293	7.0×10^{-4}
17a	P(OPh)$_3$	293	4.6×10^{-4}
17b	P(OPh)$_3$	293	4.3×10^{-4}
18a	NEt_3	293	4.7×10^{-4}
18b	NEt_3	293	4.3×10^{-4}

19a	$P(o\text{-}C_6H_4Me)_3$	273	5.1×10^{-5}
19b	$P(o\text{-}C_6H_4Me)_3$	273	3.4×10^{-5}
20a	$P(o\text{-}C_6H_4Me)_3$	293	4.7×10^{-4}
20b	$P(o\text{-}C_6H_4Me)_3$	293	5.0×10^{-4}

Table S2. The order in PPh_3 was investigated by varying amounts of PPh_3 added into each kinetic experiment. All reactions were carried out with 2-bromoethylbenzene (0.3 mol L^{-1}) as substrate in toluene-d_8.

**Entry**	**[PPh_3] (mmol L^{-1})**	**T (K)**	**k_{obs} (s^{-1})**
1a	3.3	263	4.5×10^{-4}
1b	3.3	263	4.9×10^{-4}
2a	6.6	263	1.1×10^{-3}
2b	6.6	263	1.0×10^{-3}
3a	9.9	263	1.5×10^{-3}
3b	9.9	263	1.6×10^{-3}
4a	16.5	263	2.1×10^{-3}
4b	16.5	263	2.1×10^{-3}

2. Cross-Coupling Reactions

2.1. Results from Cross-Coupling Reactions

Table S3. Duplicate results of all cross-coupling reactions carried out with 2-chloroethylbenzene. Ni(COD)(dppf) (5 mol%) was used as the catalyst for all reactions, with 5 mol% of the additive present.

Entry	Additive	4-Cl (%)	6 (%)	7 (%)	8(%)	Styrene (%)	PhEt (%)	PhPh (%)
1a	PPh_3	14	1	25	7	2	1	53
1b	PPh_3	13	1	24	7	5	3	50
2a	$FcPPh_2$	26	2	19	6	7	2	42
2b	$FcPPh_2$	28	2	20	5	8	2	38
3a	$P(o\text{-}C_6H_4Me)_3$	5	2	10	3	5	2	65
3b	$P(o\text{-}C_6H_4Me)_3$	9	2	9	4	4	2	60
4a	$P(m\text{-}C_6H_4Me)_3$	0	4	30	1	4	3	48
4b	$P(m\text{-}C_6H_4Me)_3$	0	4	29	1	6	3	44
5a	$P(p\text{-}C_6H_4Me)_3$	4	2	27	1	5	3	43
5b	$P(p\text{-}C_6H_4Me)_3$	0	4	25	1	4	3	49
6a	$P(p\text{-}C_6H_4OMe)_3$	11	3	39	12	8	4	81
6b	$P(p\text{-}C_6H_4OMe)_3$	7	2	27	8	7	4	50
7a	$P(p\text{-}C_6H_4CF_3)_3$	42	1	12	6	8	3	58
7b	$P(p\text{-}C_6H_4CF_3)_3$	43	1	11	5	5	2	60
8a	$P(p\text{-}C_6H_4F)_3$	16	1	21	9	5	3	54
8b	$P(p\text{-}C_6H_4F)_3$	19	1	19	9	5	2	57
9a	PMe_3	12	2	11	7	1	0	57
9b	PMe_3	13	2	11	7	1	2	57
10a	PBu_3	3	2	8	13	6	3	68
10b	PBu_3	0	2	10	13	4	3	75
11a	PCy_3	7	2	15	10	1	3	62
11b	PCy_3	15	2	15	7	3	3	52
12a	$P(OPh)_3$	5	2	4	10	4	4	55
12b	$P(OPh)_3$	9	3	5	7	9	3	52
13a	$AsPh_3$	5	2	4	11	4	4	65
13b	$AsPh_3$	9	3	5	12	9	3	70
14a	$SbPh_3$	28	15	5	6	3	2	72
14b	$SbPh_3$	26	14	5	6	4	3	64
15a	2,2′-bipyridine	7	1	5	8	7	0	74
15b	2,2′-bipyridine	7	3	6	9	6	2	70
16a	NEt_3	13	2	8	11	3	3	63
16b	NEt_3	7	2	10	11	8	3	68

Table S4. Duplicate results of all cross-coupling reactions carried out with 2-bromoethylbenzene. Ni(COD)(dppf) (5 mol%) was used as the catalyst for all reactions, with 5 mol% of the additive present.

Entry	Additive	4-Br (%)	6 (%)	7 (%)	8 (%)	Styrene (%)	Ethylbenzene (%)	Biphenyl (%)
1a	PPh_3	0	14	33	5	2	2	52
1b	PPh_3	0	15	31	4	4	3	43
2a	$FcPPh_2$	9	17	24	3	9	2	31
2b	$FcPPh_2$	11	17	24	3	10	2	32
3a	$P(o\text{-}C_6H_4Me)_3$	0	12	19	8	3	15	24
3b	$P(o\text{-}C_6H_4Me)_3$	0	11	16	8	3	17	26
4a	$P(m\text{-}C_6H_4Me)_3$	0	24	35	3	7	8	10
4b	$P(m\text{-}C_6H_4Me)_3$	0	28	36	2	7	5	7
5a	$P(p\text{-}C_6H_4Me)_3$	0	21	36	3	7	9	10
5b	$P(p\text{-}C_6H_4Me)_3$	0	20	34	3	4	10	9
6a	$P(p\text{-}C_6H_4OMe)_3$	0	18	34	3	6	3	38
6b	$P(p\text{-}C_6H_4OMe)_3$	0	20	34	3	2	2	44
7a	$P(p\text{-}C_6H_4CF_3)_3$	2	19	25	4	5	13	17
7b	$P(p\text{-}C_6H_4CF_3)_3$	5	17	25	4	5	8	14
8a	$P(p\text{-}C_6H_4F)_3$	0	14	29	6	2	2	52
8b	$P(p\text{-}C_6H_4F)_3$	0	13	29	6	6	3	45
9a	PMe_3	0	42	21	2	10	3	33
9b	PMe_3	0	44	20	1	6	2	35
10a	PBu_3	0	12	17	7	7	0	52
10b	PBu_3	0	16	25	10	5	0	68
11a	PCy_3	0	13	21	6	0	3	50
11b	PCy_3	2	12	17	4	6	2	29
12a	$P(OPh)_3$	0	28	27	3	10	3	38
12b	$P(OPh)_3$	0	28	29	3	9	2	45
13a	$AsPh_3$	0	6	13	9	3	3	70
13b	$AsPh_3$	0	4	15	8	8	3	48
14a	$SbPh_3$	0	14	25	4	10	3	54
14b	$SbPh_3$	0	14	26	4	10	3	53
15a	2,2′-bipyridine	0	6	11	5	11	4	50
15b	2,2′-bipyridine	0	8	14	6	9	3	67
16a	NEt_3	0	10	20	8	10	3	51
16b	NEt_3	0	8	17	5	8	3	33

Table S5. Duplicate results of all cross-coupling reactions carried out with 2-iodoethylbenzene. Ni(COD)(dppf) (5 mol%) was used as the catalyst for all reactions, with 5 mol% of the additive present.

Entry	Additive	4-I (%)	6 (%)	7 (%)	8 (%)	Styrene (%)	Ethylbenzene (%)	Biphenyl (%)
1a	PPh_3	0	59	5	1	10	8	15
1b	PPh_3	0	48	2	1	9	15	21
2a	$FcPPh_2$	8	40	2	1	10	2	31
2b	$FcPPh_2$	9	40	2	1	8	2	32
3a	P(*o*-$C_6H_4Me)_3$	0	46	3	1	6	14	19
3b	P(*o*-$C_6H_4Me)_3$	0	49	3	1	8	14	17
4a	P(*m*-$C_6H_4Me)_3$	0	56	7	1	8	4	11
4b	P(*m*-$C_6H_4Me)_3$	0	59	6	1	8	5	9
5a	P(*p*-$C_6H_4Me)_3$	0	55	6	1	5	7	12
5b	P(*p*-$C_6H_4Me)_3$	0	54	7	1	7	7	12
6a	P(*p*-$C_6H_4OMe)_3$	0	58	6	1	7	4	8
6b	P(*p*-$C_6H_4OMe)_3$	0	59	6	1	6	6	12
7a	P(*p*-$C_6H_4CF_3)_3$	0	50	7	1	8	3	14
7b	P(*p*-$C_6H_4CF_3)_3$	0	53	7	1	7	4	10
8a	P(*p*-$C_6H_4F)_3$	0	56	7	1	8	5	11
8b	P(*p*-$C_6H_4F)_3$	0	60	6	1	6	5	12
9a	PMe_3	0	54	5	1	10	3	44
9b	PMe_3	0	51	5	1	8	3	43
10a	PBu_3	0	50	2	0	5	12	14
10b	PBu_3	0	50	2	0	4	17	16
11a	PCy_3	0	59	4	1	8	16	21
11b	PCy_3	0	59	6	1	8	10	18
12a	$P(OPh)_3$	0	53	6	2	12	4	39
12b	$P(OPh)_3$	0	49	5	2	9	3	40
13a	$AsPh_3$	0	39	4	1	12	4	49
13b	$AsPh_3$	0	38	4	1	10	4	51
14a	$SbPh_3$	0	22	6	3	10	3	51
14b	$SbPh_3$	0	21	6	3	12	4	50
15a	2,2′-bipyridine	0	45	4	1	10	3	49
15b	2,2′-bipyridine	0	44	4	1	10	4	45
16a	NEt_3	12	31	1	1	11	3	32
16b	NEt_3	8	33	1	2	11	3	35

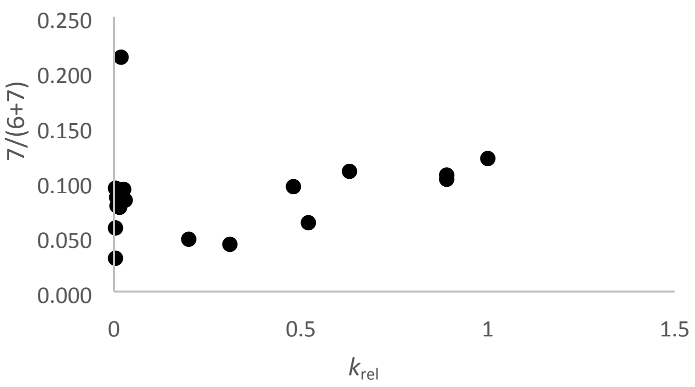

Figure S1. Ratio of products vs k_{rel} for iodoethylbenzene.

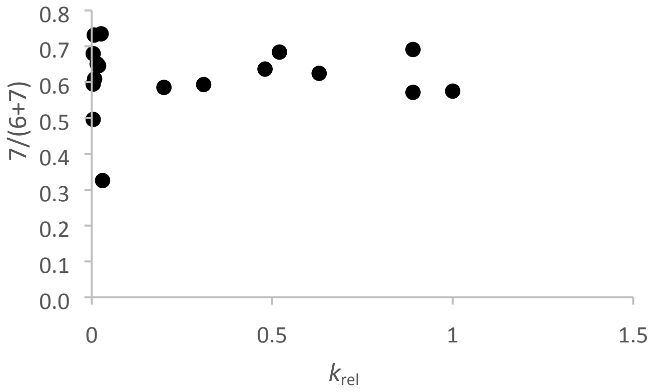

Figure S2. Ratio of products vs k_{rel} for bromoethylbenzene.

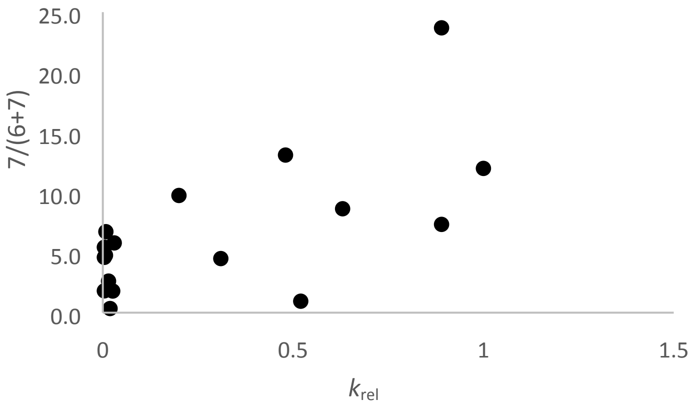

Figure S3. Ratio of products vs k_{rel} for chloroethylbenzene.

2.2. GC-FID Calibrations

Conversions in both cross-couplings and product studies stated are in reference to an internal standard (*n*-tetradecane). All substrates and any products were calibrated in reference to *n*-tetradecane. All analysis was determined *via* a GC-FID and the specific method involved a temperature profile as follows: 40 °C per minute for 5 minutes, followed by 20 °C per minute for 5 minutes, up to 320 °C.

An example of how to calibrate for a material is shown below.

i. An accurately known amount of material (to be calibrated) was weighed into a medium sized vial (roughly 10 mg).
ii. A different, accurately known mass of *n*-tetradecane was put into each vial (approximately 10, 20, 50, 100, 250, 500 and 1000 mg).
iii. These mixtures were diluted in chloroform and a sample of each one was analysed by GC-FID.
iv. The areas of the peaks corresponding to both the substrate in question and *n*-dodecane were measured.
v. The ratios of the mmol of compound/mmol *n*-tetradecane vs peak area of substrate/peak area of *n*-dodecane were plotted (examples in Table S10, Figure S9).
vi. The graph produced gave a straight line with an $R^2 > 0.99$, the gradient of this line was the response factor of the substrate being calibrated for.

This response factor is then taken into account through calculations when reactions have taken place – thus allowing the determination of the amount in the product mixtures.

Table S6. Response factors given against standards for the substrates/products used in this work.

**Substrate**	***Response factor against n*-tetradecane**
(2-iodoethyl)benzene	0.5209
(2-bromoethyl)benzene	0.5757
(2-chloroethyl)benzene	0.4423
1,2-diphenylethane	0.8814
1,1-diphenylethane	1.0273
2,3-diphenylbutane	1.1518
1,4-diphenylbutane	0.8355
ethylbenzene	0.4636
styrene	0.5079
biphenyl	0.7207

3. NMR Spectra

1,4-Diphenylbutane

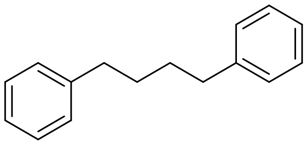

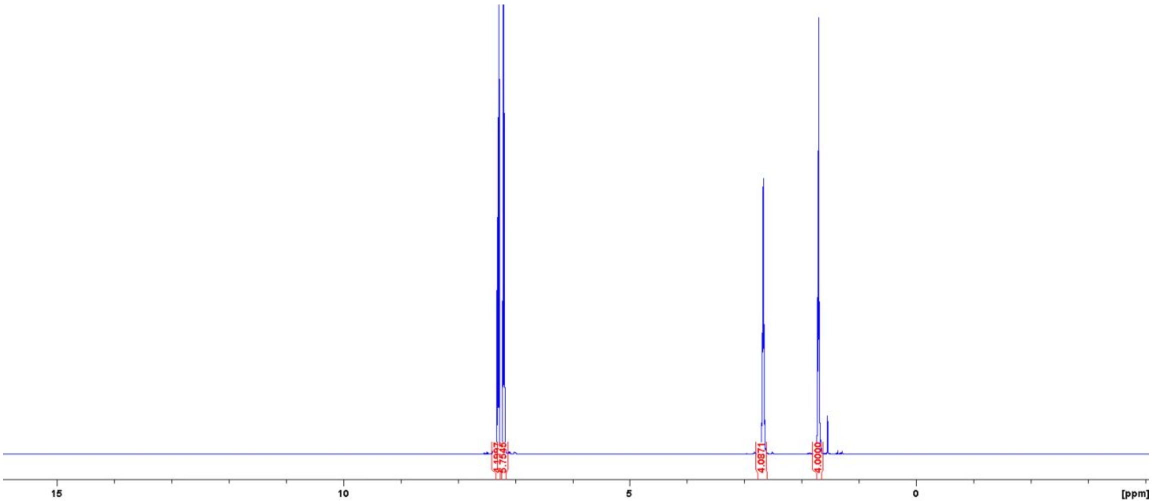

Figure S4. ^{1}H NMR of 1,4-diphenylbutane in $CDCl_3$.

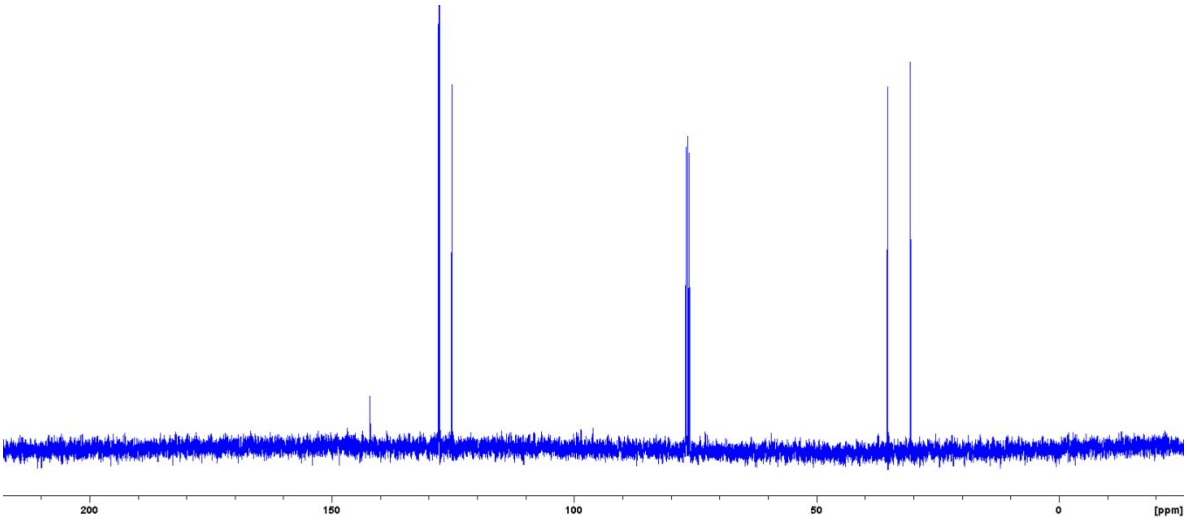

Figure S5. $^{13}C\{^{1}H\}$ NMR of 1,4-diphenylbutane in $CDCl_3$.

2,3-Diphenylbutane

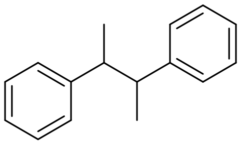

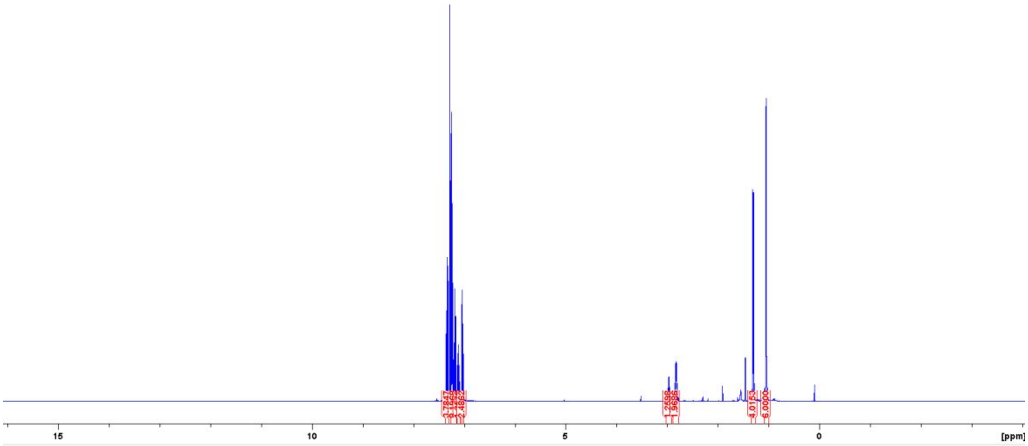

Figure S6. ^{1}H NMR of 2,3-diphenylbutane in $CDCl_3$.

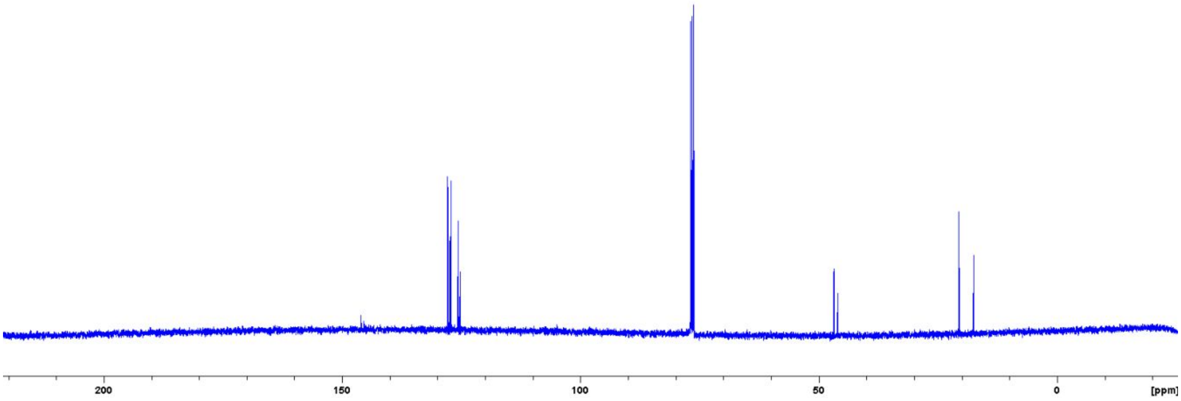

Figure S7. $^{13}C\{^{1}H\}$ NMR of 2,3-diphenylbutane in $CDCl_3$.

Diphenylphosphinoferrocene

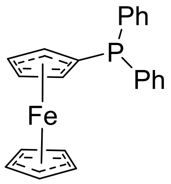

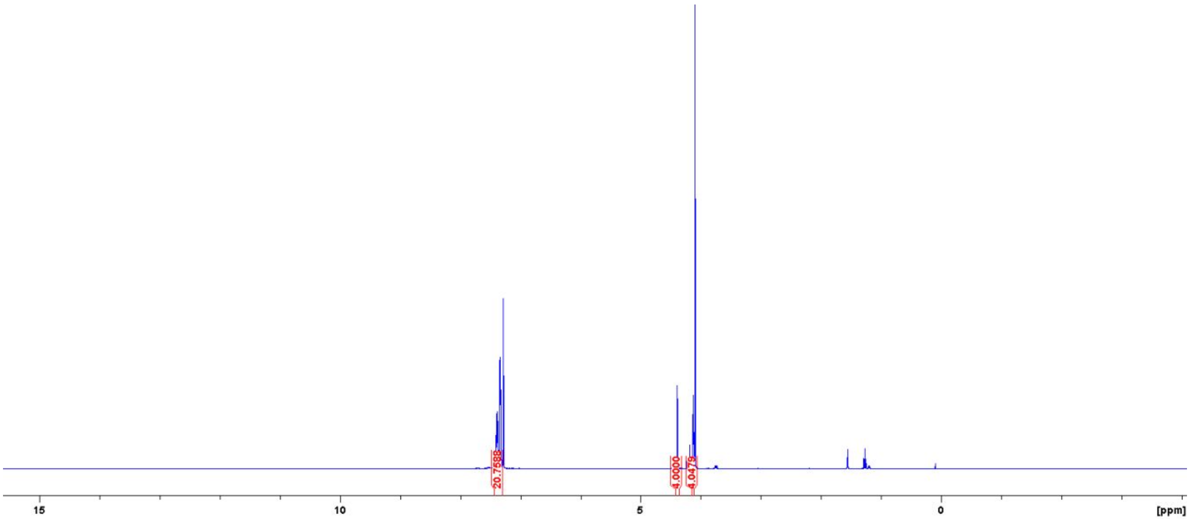

Figure S8. ^{1}H NMR of diphenylphosphinoferrocene *in $CDCl_3$*.

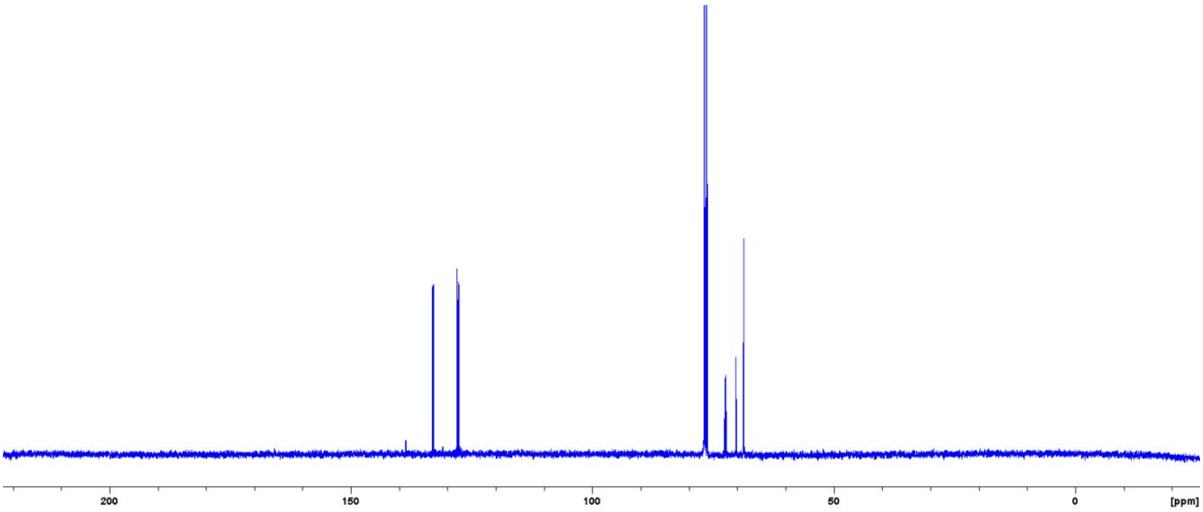

Figure S9. $^{13}C\{^{1}H\}$ NMR of diphenylphosphinoferrocene in $CDCl_3$.

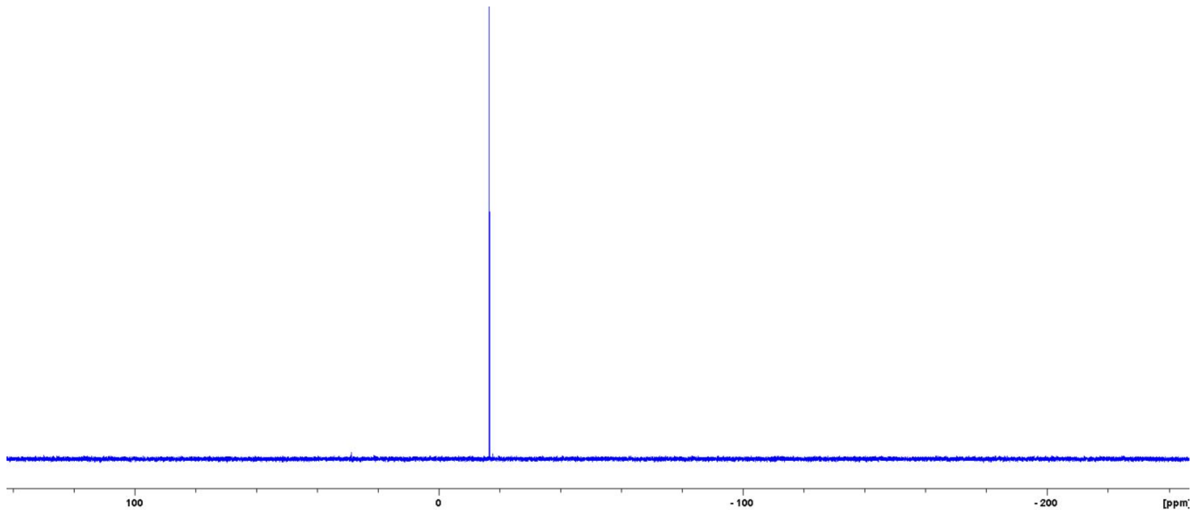

Figure S10. $^{31}P\{^{1}H\}$ NMR of diphenylphosphinoferrocene in $CDCl_3$.

4. Kinetic Plots

The plots below record data for each experiment in tables S1-S2.

4.1. Data for Table S1

Entry 1a

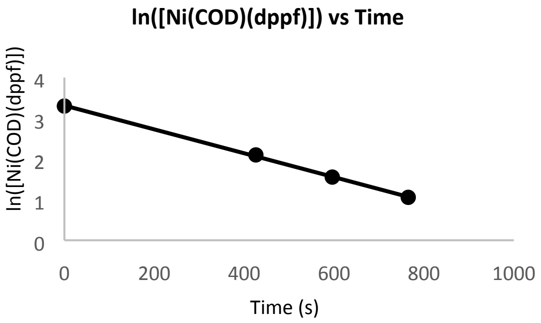

Entry 1b

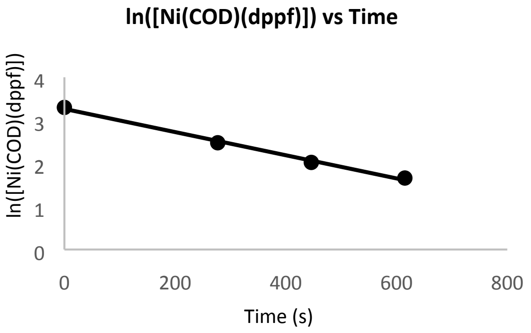

Entry 2a

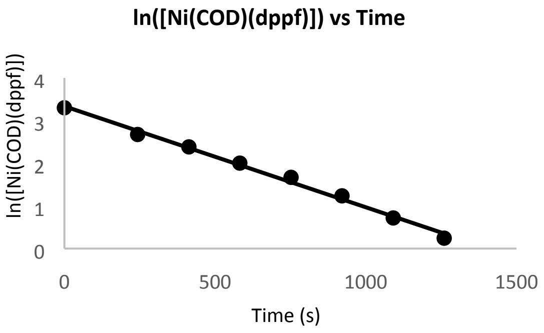

Entry 2b

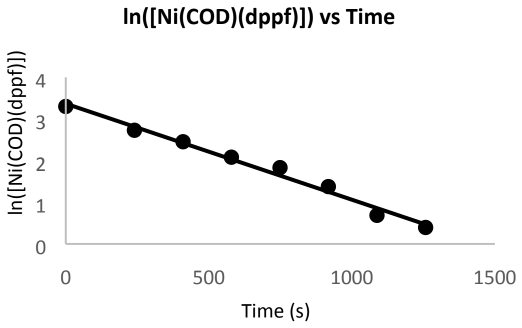

Entry 3a

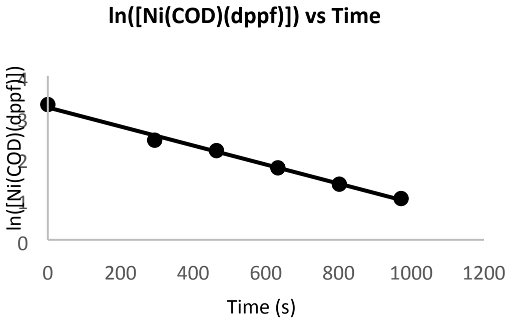

Entry 3b

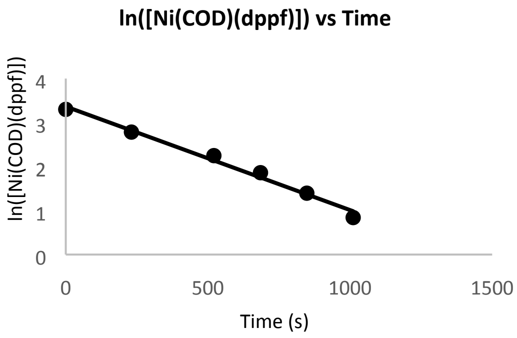

Entry 4a

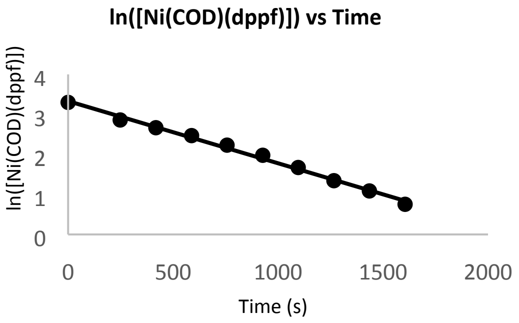

Entry 5b

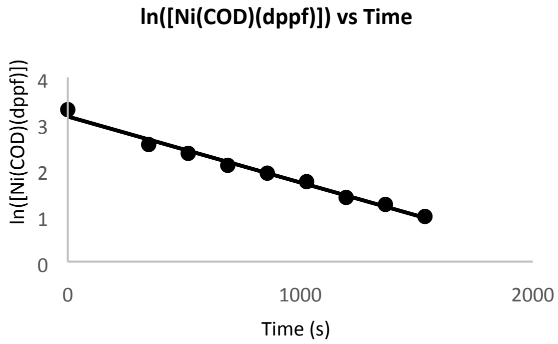

Entry 4b

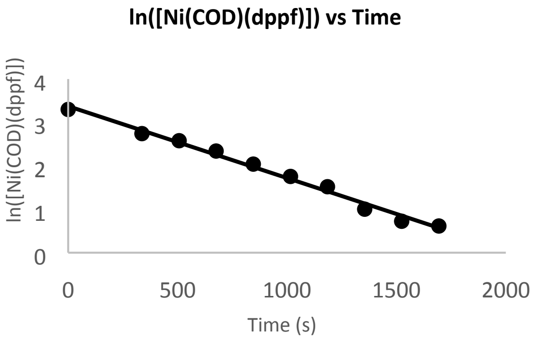

Entry 6a

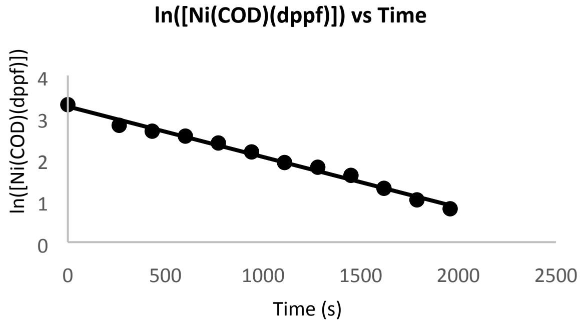

Entry 5a

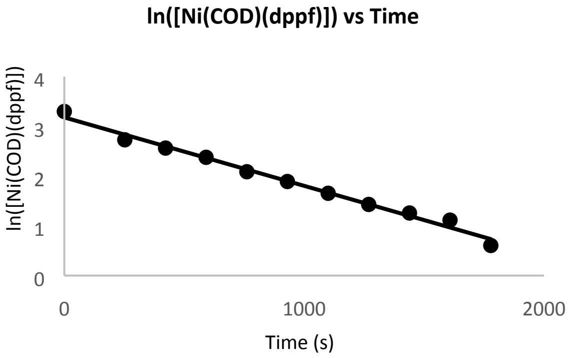

Entry 6b

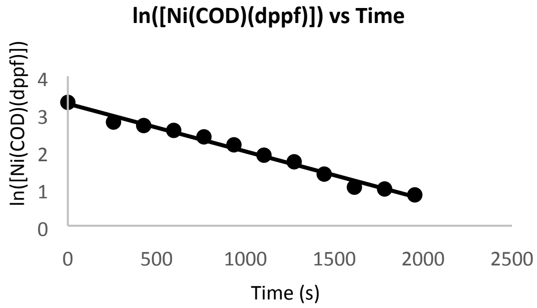

Entry 7a

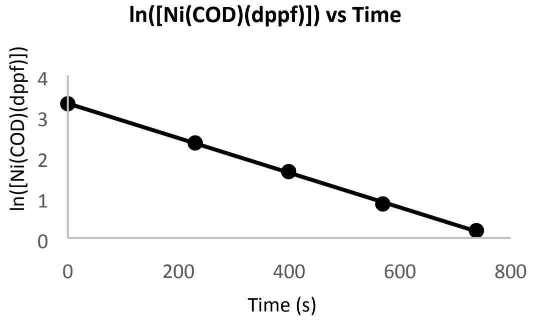

Entry 7b

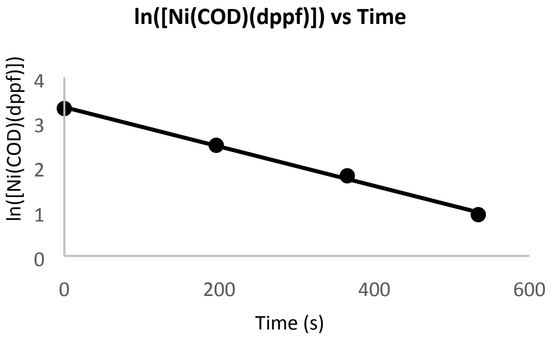

Entry 9b

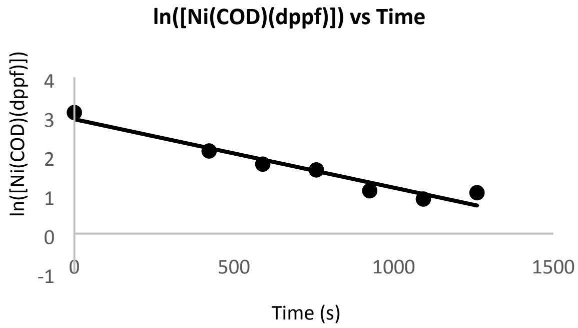

Entry 8a

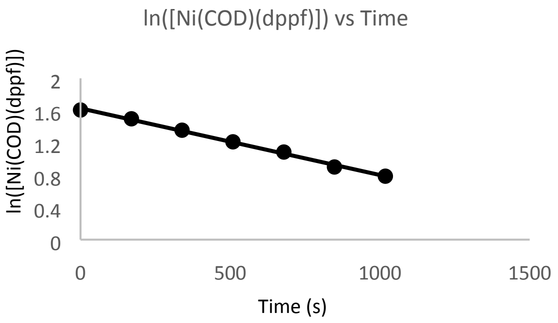

Entry 10a

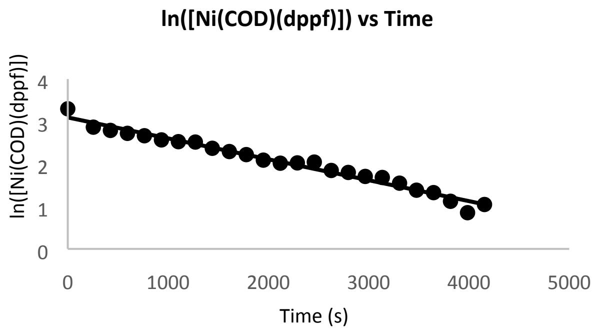

Entry 8b

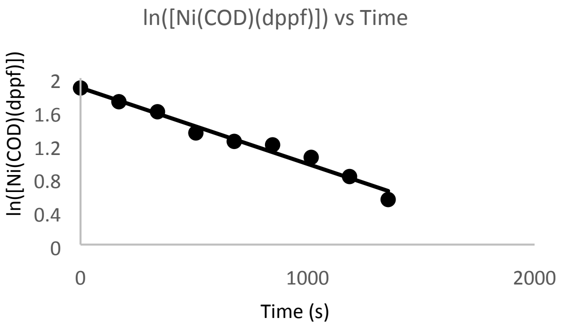

Entry 10b

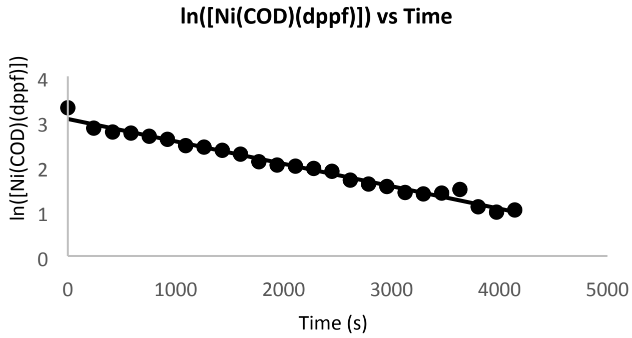

Entry 9a

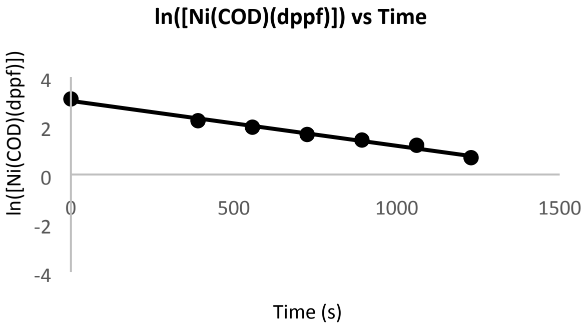

Entry 11a

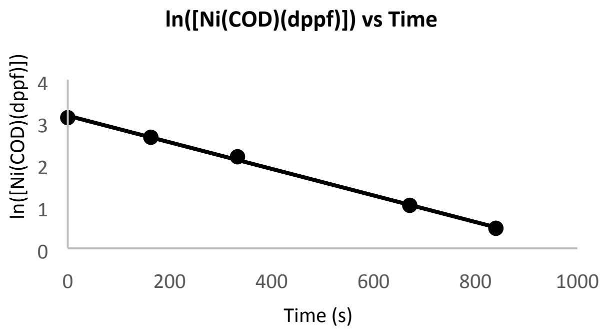

Entry 11b

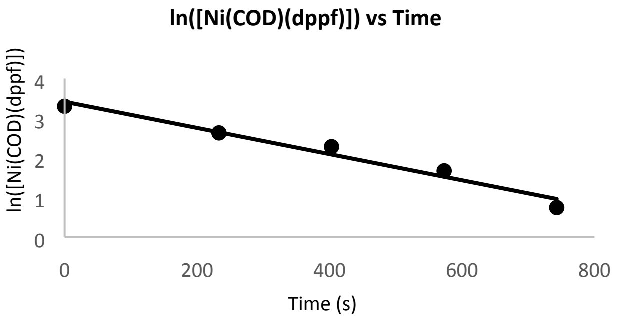

Entry 12a

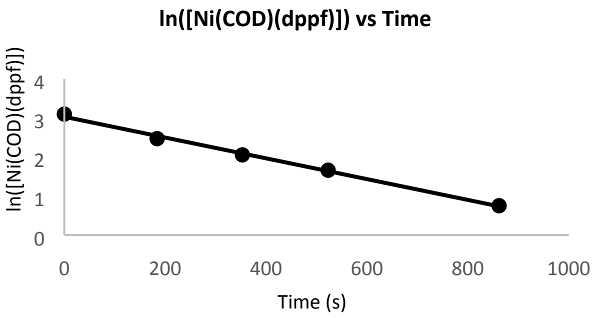

Entry 12b

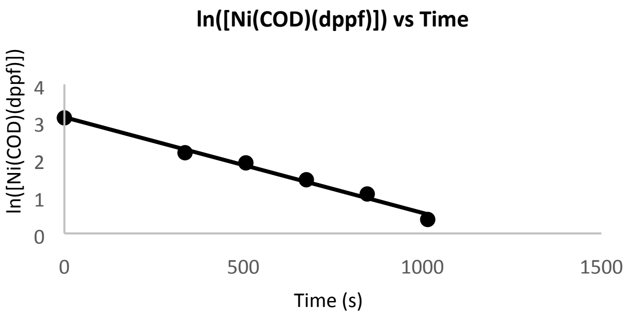

Entry 13a

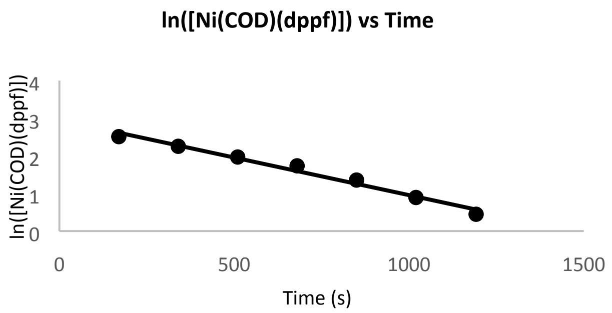

Entry 13b

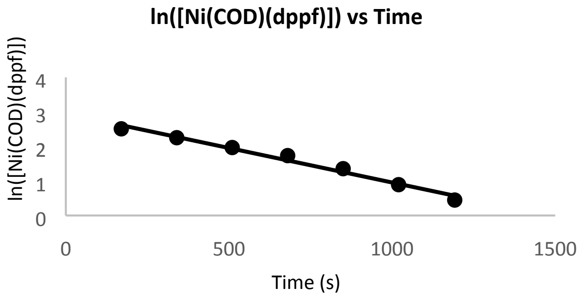

Entry 14a

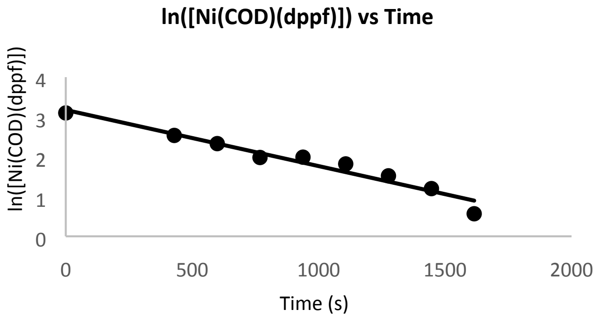

Entry 14b

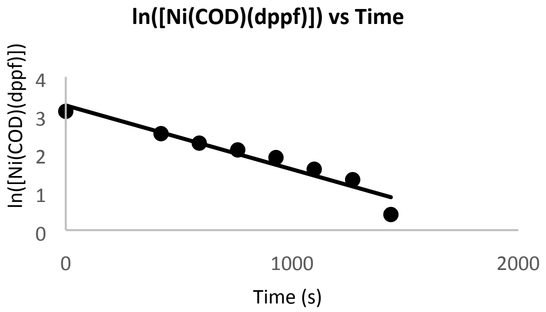

Entry 15a

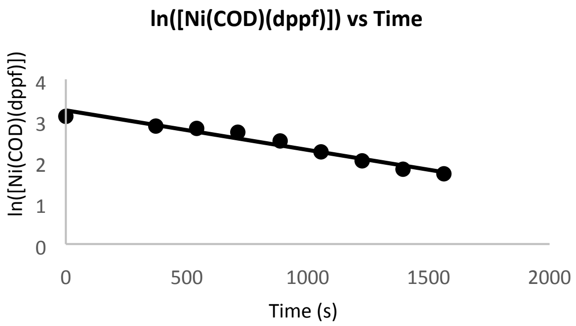

Entry 15b

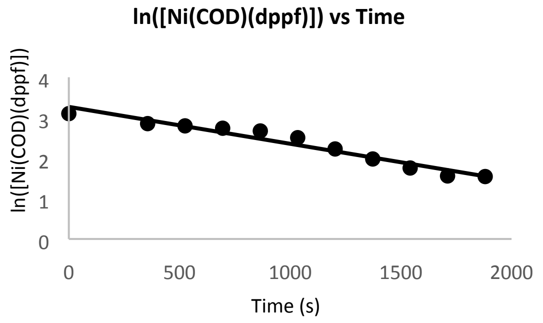

Entry 17b

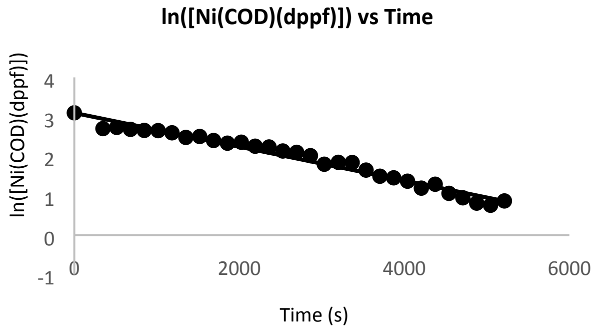

Entry 16a

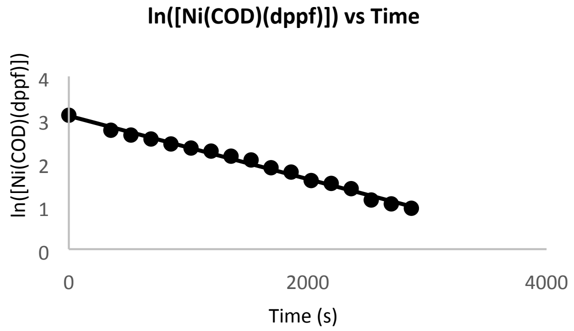

Entry 18a

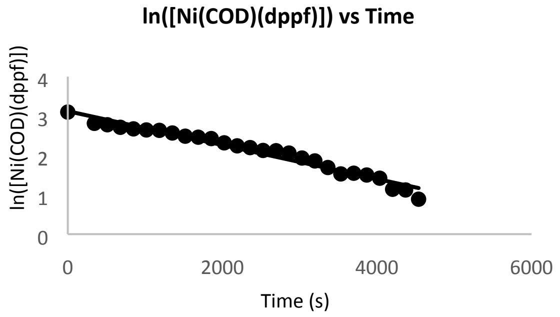

Entry 16b

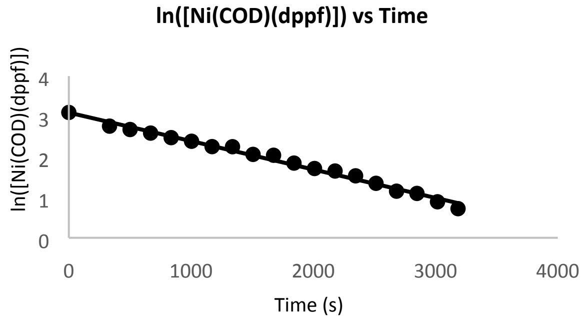

Entry 18b

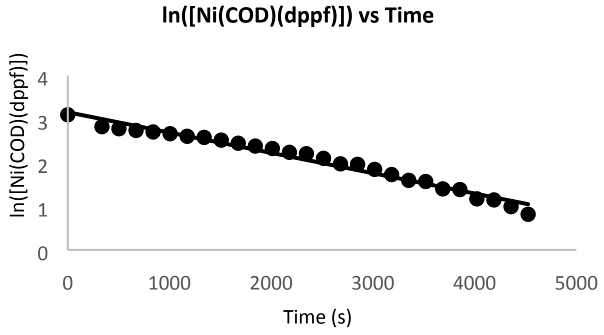

Entry 17a

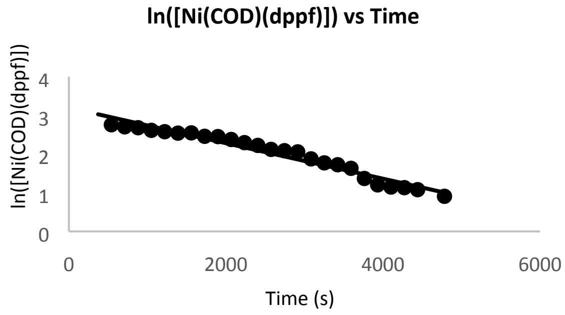

Entry 19a

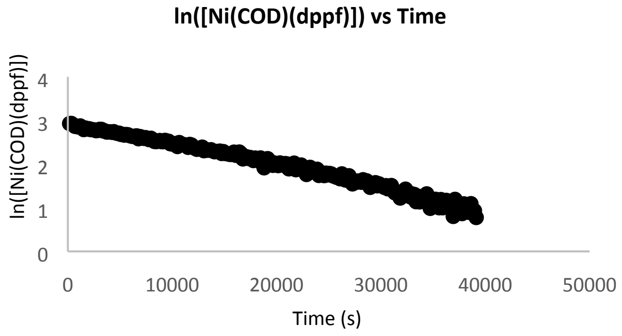

Entry 19b

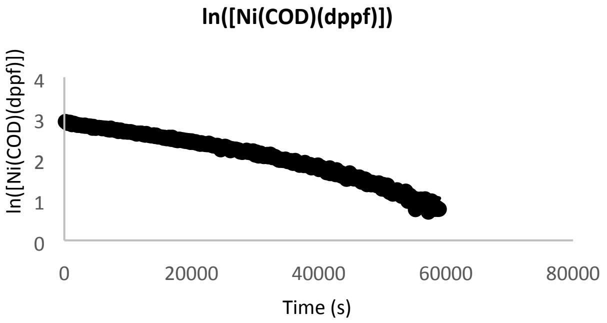

Entry 20a

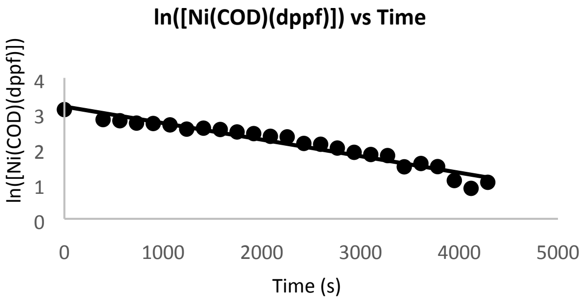

Entry 20b

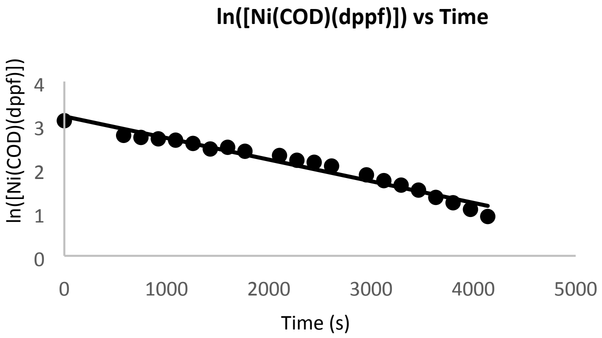

6.2. Data for Table S2

Entry 1a

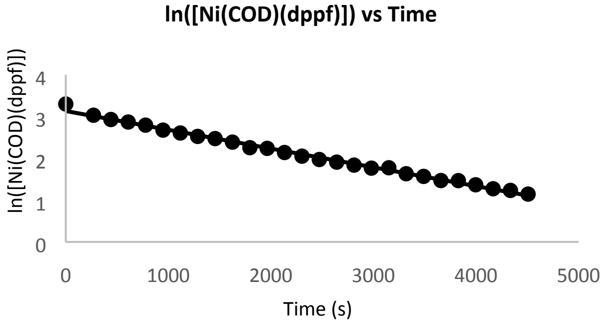

Entry 1b

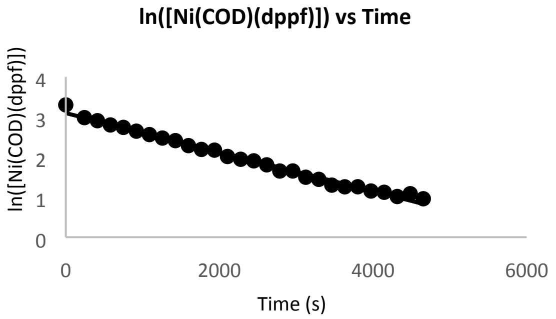

Entry 2a

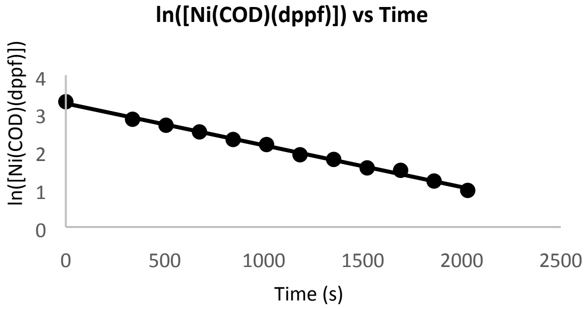

Entry 2b

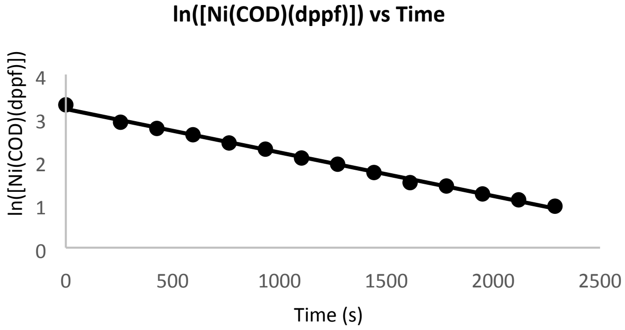

Entry 3a

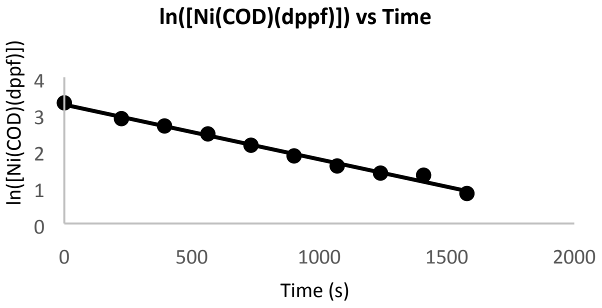

Entry 4a

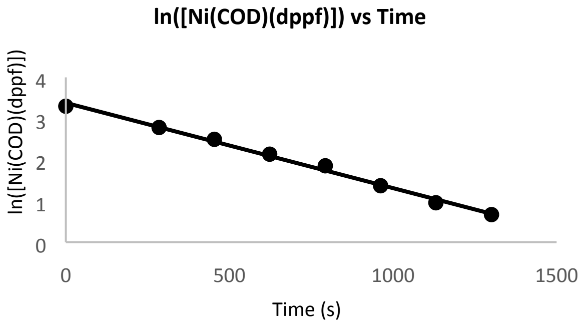

Entry 3b

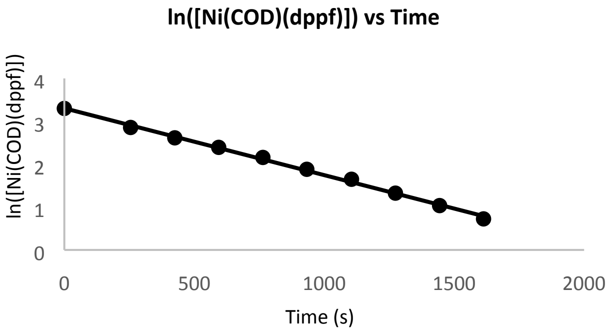

Entry 4b

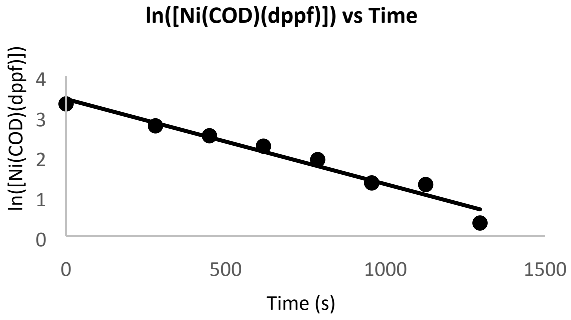

5. Computational Chemistry

5.1 Methodology

Software. All DFT calculations were carried out using Gaussian16 Rev. A.03.[8] All DLPNO-CCSD(T) calculations[9-11] were carried out using Orca 4.2.1.[12,13]

Optimisation. All geometry optimisations were carried out using the B3LYP functional[14-17] with the Grimme D3 empirical dispersion correction.[18] The LANL2TZ(f) pseudopotential/ECP was used for nickel and iron, while LANL2DZ(d,p) was used for bromine, arsenic, and antimony.[19-21] The 6-31G(d) basis set was used for all other atoms. Optimisations were carried out without symmetry constraints in the gas phase. Frequency calculations were used to verify the nature of stationary points (zero imaginary frequencies for each of the minima; one imaginary frequency for each of the transition states). Each transition state was checked using IRC calculations or by optimisations along the reaction coordinate.

Single Points. Unless otherwise stated, the energies of each of the structure were calculated using single point calculations with the M06 functional,[22] the LANLDZ(d,p) pseudopotential/ECP on antimony, and the 6-311+G(d,p) basis set on all other atoms. Solvation was modelled using the SMD model (in benzene).[23]

Corrections. The free energy of each species was corrected for solution-phase behaviour by adding 1.89 kcal/mol.[24] The electronic energies of each open shell singlet species was corrected for triplet contamination using Yamaguchi's approach:[25]

$$E_{corr.\,OSS} = \frac{2E_{OSS} - \left(E_{triplet} \cdot \langle S^2 \rangle_{OSS}\right)}{2 - \langle S^2 \rangle_{OSS}}$$

Free Energies. All energies quoted in the manuscript are free energies in kcal/mol with respect to [Ni(COD)(dppf)].

5.2 %V_{bur} Calculations for L in [Ni(dppf)(L)]

All buried volume calculations were carried out using SambVca v2.1, which can be accessed for free *via* the internet at https://www.molnac.unisa.it/OMtools/sambvca2.1/index.html. Further details about this tool can be found in the relevant publications.[26-28]

Table S7. Percent buried volume for ligands L in DFT-derived [Ni(dppf)(L)] complexes.

**Ligand**	**%V_{bur}**	**Ligand**	**%V_{bur}**
PMe_3	24.0	PPh_3	31.2
PCy_3	31.9	(*p*-$MeOC_6H_4)_3P$	31.3
$AsPh_3$	22.7	(*p*-$MeC_6H_4)_3P$	31.3
$SbPh_3$	27.7	(*p*-$FC_6H_4)_3P$	31.4
		(*p*-$F_3CC_6H_4)_3P$	31.3

5.3 Benchmarking Studies

Benchmarking was carried out using the published geometries for [Ni(COD)(dppf)], COD, and [Ni(dppf)$_2$] obtained at the B3LYP-D3/6-31G(d)+LANL2DZ(d,p)+LANL2TZ(f) level of theory in the gas phase. A new geometry was obtained for dppf which is typically *ca.* 1- 2 kcal/mol lower in energy depending on the level of theory used.

Single point calculations were carried out using different functionals, basis sets, grids, and dispersion corrections. Benzene solvation (SMD model) was applied in each case. Free energy changes were obtained by applying the correction to free energy obtained during the geometry optimisation for each structure (which included a frequency calculation).

Table S8. Correction to free energy applied to each electronic energy obtained.

**Species**	[Ni(COD)(dppf)]	dppf	COD	[Ni(dppf)$_2$]
**G_{corr} (Ha)**	0.631292	0.452033	0.149935	0.946725

DLPNO-CCSD(T) using the cc-pVTZ basis set, and TIGHTSCF and RIJCOSX options. ΔG = -3.8 kcal/mol.

Table S9. Electronic energies obtained using DLPNO-CCSD(T)/cc-pVTZ calculations.

**Species**	[Ni(COD)(dppf)]	dppf	COD	[Ni(dppf)$_2$]
**E (Ha)**	-5074.026353	-3254.895329	-311.444404	-8017.493088

DFT. Electronic energies in Ha. Free energy change in in kcal/mol.

Table S10. Electronic energies obtained using DFT calculations.

**Functional**	**Basis Set**	**Disp. Corr.**	**Grid**	**[Ni(COD)(dppf)]**	**dppf**	**[Ni(dppf)$_2$]**	**COD**	**ΔG**
B3LYP	6-311+G(d,p)	D3	UltraFine	-5079.936888	-3259.399194	-8027.22525	-312.1423956	-11.9
B3LYP	6-311+G(d,p)	D3	SuperFine	-5079.936883	-3259.399194	-8027.225207	-312.1423932	-11.9
B3LYP	6-311+G(d,p)	D3BJ	UltraFine	-5080.065996	-3259.499706	-8027.446896	-312.1604942	-17.9
B3LYP	6-311+G(2d,2p)	D3	UltraFine	-5080.005417	-3259.453800	-8027.336275	-312.1550359	-12.2
B3LYP	6-311+G(2d,2p)	XDM[a]	UltraFine	-5080.097799	-3259.529563	-8027.480728	-312.1668943	- 5.2
CAM-B3LYP	6-311+G(d,p)	-	UltraFine	-5078.888514	-3258.552238	-8025.497539	-311.9309316	17.5
CAM-B3LYP	6-311+G(d,p)	D3	UltraFine	-5078.999192	-3258.617940	-8025.699386	-311.9417672	- 7.2
CAM-B3LYP	6-311+G(d,p)	D3BJ	UltraFine	-5079.043832	-3258.655317	-8025.7848	-311.9466062	-12.1
BP86	6-311+G(d,p)	D3	UltraFine	-5080.436661	-3259.642782	-8027.992979	-312.1269391	-17.8
BP86	6-311+G(d,p)	D3BJ	UltraFine	-5080.543627	-3259.726679	-8028.18413	-312.1398999	-25.4
M06	6-311+G(d,p)	-[b]	UltraFine	-5078.288467	-3258.130334	-8024.572644	-311.8618736	- 1.9
M06	6-311+G(d,p)	D3	UltraFine	-5078.319811	-3258.149504	-8024.636856	-311.8640859	-12.7
M06-L	6-311+G(d,p)	-[b]	UltraFine	-5079.44428	-3259.057408	-8026.453381	-312.0715822	- 7.1
M11	6-311+G(d,p)	-[b]	UltraFine	-5078.343213	-3258.275085	-8024.728845	-311.9027729	- 0.9
M11-L	6-311+G(d,p)	-[b]	UltraFine	-5079.673051	-3259.151228	-8026.775909	-312.052852	4.7
MN15	6-311+G(d,p)	-[b]	UltraFine	-5078.092054	-3257.676285	-8024.082175	-311.7037077	- 3.9
ωB97X-D	6-311+G(d,p)	(D2)[c]	UltraFine	-5079.246492	-3258.838687	-8026.089777	-312.021771	- 9.2

[a] Single point calculation carried out without dispersion correction, and XDM correction added subsequently using postg.

[b] M06(-L), M11(-L), and MN15 include dispersion treaments in the functional.

[c] ωB97X-D includes the D2 empirical correction.

5.4 Table of Energies

Small basis set (sb): B3LYP-D3/6-31G(d)+LANL2TZ(f)[Ni,Fe]+LANL2DZ(d,p)[Br,As,Sb]. Large basis set (lb): M06/6-311+G(d,p)+LANL2DZ(d,p)[Sb] in benzene solvent (SMD). G' refers to the free energy with a 1.89 kcal/mol correction to reflect solution behaviour.

Table S11. Energies of species modelled during this study.

Species	Hartrees			kcal/mol		
	H_{corr} (sb)	G_{corr} (sb)	E (lb)	G (lb)	G' (lb)	G'_{rel} (lb)
COD	0.189706	0.149935	-311.861874	-195602.2	-195600.3	n/a
[Ni(COD)(dppf)]	0.745713	0.631292	-5078.288467	-3186278.0	-3186276.1	0.0
$PhCH_2CH_2Br$	0.157776	0.112576	-2884.131038	-1809748.9	-1809747.0	n/a
Ligands						
dppf						n/a
$FcPPh_2$	0.365019	0.292357	-2454.324035	-1539928.1	-1539926.2	n/a
PPh_3	0.291287	0.227957	-1035.919675	-649906.4	-649904.5	n/a
(p-$MeOC_6H_4)_3P$	0.397711	0.316846	-1379.401699	-865388.8	-865386.9	n/a
(p-$MeC_6H_4)_3P$	0.379656	0.301784	-1153.805875	-723834.7	-723832.9	n/a
(p-$FC_6H_4)_3P$	0.269185	0.199627	-1333.642568	-836748.1	-836746.2	n/a
(p-$F_3CC_6H_4)_3P$	0.316592	0.222625	-2047.020929	-1284385.3	-1284383.4	n/a
PMe_3	0.121050	0.084159	-461.0186095	-289240.7	-289238.8	n/a
PCy_3	0.507516	0.440703	-1046.796141	-656598.0	-656596.1	n/a
$P(OPh)_3$	0.306454	0.232962	-1261.607505	-791524.5	-791522.6	n/a
NMe_3	0.127619	0.094124	-174.3859669	-109369.8	-109367.9	n/a
$AsPh_3$	0.290176	0.224982	-2930.342451	-1898676.5	-1838674.6	n/a
$SbPh_3$	0.289430	0.222519	-699.9520144	-439086.9	-439085.0	n/a
[Ni(dppf)(L)] closed						
dppf	1.108881	0.946725	-8024.572644	-5034901.3	-5034899.4	-1.5
[Ni(dppf)(L)]						
dppf	1.106920	0.932720	-8024.556589	-5034900.0	-5034898.1	-0.2
$FcPPh_2$	0.921452	0.775175	-7220.751568	-4530603.6	-4530601.7	0.3
PPh_3	0.847428	0.708021	-5802.342713	-3640580.7	-3640578.8	1.4
(p-$MeOC_6H_4)_3P$	0.953760	0.796793	-6415.825276	-3856063.6	-3856061.7	1.0
(p-$MeC_6H_4)_3P$	0.935762	0.782215	-5920.228960	-3714508.9	-3714507.0	1.6
(p-$FC_6H_4)_3P$	0.825399	0.680476	-6100.067072	-3827422.9	-3827421.0	1.0
(p-$F_3CC_6H_4)_3P$	0.872851	0.704474	-6813.448079	-4275061.2	-4275059.3	0.0
PMe_3	0.676870	0.558751	-5227.438417	-3279916.5	-3279914.6	0.0
PCy_3	1.063702	0.919943	-5813.221007	-3647274.0	-3647272.1	-0.2
$P(OPh)_3$	0.863113	0.718871	-6028.048362	-3782206.4	-3782204.5	-6.1
NMe_3	0.683937	0.569519	-4940.783842	-3100031.3	-3100029.4	14.3
$AsPh_3$	0.846256	0.703816	-7696.764277	-4829350.9	-4829349.0	1.4
$SbPh_3$	0.845310	0.698543	-5466.371879	-3429761.8	-3429759.9	0.9
[Ni(dppf)(L)$_2$]						
$FcPPh_2$	1.292040	1.106202	-9675.099046	-6070522.2	-6070520.3	8.0
PPh_3	1.143577	0.974714	-6838.291875	-4290481.3	-4290479.4	5.3
(p-$MeOC_6H_4)_3P$	1.356503	1.153028	-7525.259803	-4721448.3	-4721446.4	3.2
(p-$MeC_6H_4)_3P$	1.320285	1.122863	-7074.064105	-4438337.6	-4438335.7	5.8
(p-$FC_6H_4)_3P$	1.099476	0.918065	-7433.741562	-4664167.2	-4664165.3	2.9
(p-$F_3CC_6H_4)_3P$	1.194809	0.971609	-8860.503850	-5559440.4	-5559438.5	4.1
PMe_3	0.801236	0.672069	-5688.502672	-3569167.6	-3569165.7	-12.2
$P(OPh)_3$	1.173368	0.990996	-7289.716752	-4573744.5	-4573742.6	-21.6
$AsPh_3$	1.140671	0.964105	-10627.149690	-6668032.1	-6668030.2	-5.3
$SbPh_3$	1.138083	0.956712	-6166.378149	-3868862.2	-3868860.4	-14.6

Species	Hartrees			kcal/mol		
	H_{corr} (sb)	G_{corr} (sb)	E (lb)	G (lb)	G' (lb)	G'_{rel} (lb)
[Ni(dppf)(L)(BrCH$_2$CH$_2$Ph)]						
dppf	1.267529	1.072599	-10908.706954	-6844643.9	-6844642.0	2.9
$FcPPh_2$	1.081645	0.908485	-10104.896082	-6340347.9	-6340346.1	3.0
PPh_3	1.007630	0.844532	-8686.492925	-5450326.7	-5450324.8	2.5
(*p*-$MeOC_6H_4)_3P$	1.114377	0.936373	-9029.975231	-5665807.4	-5665805.5	4.2
(*p*-$MeC_6H_4)_3P$	1.096094	0.919376	-8804.378083	-5524253.7	-5524251.9	3.8
(*p*-$FC_6H_4)_3P$	0.985787	0.817695	-8984.216844	-5637168.1	-5637166.2	2.8
(*p*-$F_3CC_6H_4)_3P$	1.033205	0.845944	-9697.598125	-6084803.9	-6084802.0	4.3
PMe_3	0.837269	0.698279	-8111.584672	-5089658.1	-5089656.2	5.5
PCy_3	1.224269	1.054295	-8697.363221	-5457016.2	-5457014.4	4.5
$P(OPh)_3$	1.023321	0.855244	-8912.195805	-5591950.6	-5591948.7	-3.3
NMe_3	0.843972	0.706595	-7824.925484	-4909771.5	-4909769.6	21.1
$AsPh_3$	1.007183	0.845009	-10580.903828	-6639087.1	-6639085.3	12.1
$SbPh_3$	1.006066	0.840014	-8350.515304	-5239500.4	-5239498.5	9.3
Halide Abstraction TS			**E (lb) (singlet)**	**<S**2>**	**E (lb) (triplet)**	
dppf			-10908.670876	0.3933	-10908.652472	
$FcPPh_2$			-10104.862313	0.0054	-10104.850193	
PPh_3			-8686.457157	0	-8686.447727	
(*p*-$MeOC_6H_4)_3P$			-9029.945053	0	-9029.934611	
(*p*-$MeC_6H_4)_3P$			-8804.348658	0.0021	-8804.338545	
(*p*-$FC_6H_4)_3P$			-8984.183938	0.0028	-8984.174000	
(*p*-$F_3CC_6H_4)_3P$			-9697.565479	0.0001	-9697.555967	
PMe_3			-8111.556054	0	-8111.538071	
PCy_3			-8697.329811	0.4782	-8697.318521	
$P(OPh)_3$			-8912.158617	0.5247	-8912.146136	
NMe_3			-7824.907519	0	-7824.889909	
$AsPh_3$			-10580.881219	0.4996	-10580.863419	
$SbPh_3$			-8350.491383	0.5453	-8350.475997	

Species	Hartrees			kcal/mol		
	H_{corr} (sb)	G_{corr} (sb)	E (lb)	G (lb)	G' (lb)	G'_{rel} (lb)
Halide Abstraction TS (corrected open shell singlet)						
dppf	1.265102	1.075535	-10908.675381	-6844622.2	-6844620.4	24.6
$FcPPh_2$	1.079112	0.909312	-10104.862346	-6340326.3	-6340324.4	24.7
PPh_3	1.005314	0.843940	-8686.457157	-5450304.6	-5450302.7	24.6
(*p*-$MeOC_6H_4)_3P$	1.111627	0.933625	-9029.945053	-5665790.2	-5665788.3	21.4
(*p*-$MeC_6H_4)_3P$	1.093609	0.920037	-8804.348669	-5524234.9	-5524233.0	22.7
(*p*-$FC_6H_4)_3P$	0.983116	0.816129	-8984.183952	-5637148.4	-5637146.5	22.5
(*p*-$F_3CC_6H_4)_3P$	1.303492	0.844477	-9697.565479	-6084784.3	-6084782.4	23.8
PMe_3	0.834559	0.696342	-8111.556064	-5089641.3	-5089639.4	22.2
PCy_3	1.222399	1.059183	-8697.333359	-5456994.4	-5456992.5	26.3
$P(OPh)_3$	1.020767	0.857743	-8912.163057	-5591928.5	-5591926.6	18.8
NMe_3	0.841873	0.707226	-7824.907519	-4909759.8	-4909757.9	32.8
$AsPh_3$	1.004223	0.844126	-10580.887146	-6639077.2	-6639075.3	22.0
$SbPh_3$	1.003350	0.837213	-8350.497150	-5239490.7	-5239488.8	19.0
[NiBr(dppf)(L)] + PhCH$_2$CH$_2^·$			**E (lb) (singlet)**	**<S**2>**	**E (lb) (triplet)**	
dppf			-10908.693066	1.0229	-10908.693046	
$FcPPh_2$			-10104.888326	1.0213	-10104.888250	
PPh_3			-8686.473471	1.0233	-8686.482661	
(*p*-$MeOC_6H_4)_3P$			-9029.966171	1.0262	-9029.966171	
(*p*-$MeC_6H_4)_3P$			-8804.358643	1.0242	-8804.368738	

(*p*-FC$_6$H$_4$)$_3$P			-8984.204510	1.0251	-8984.204498	
(*p*-F$_3$CC$_6$H$_4$)$_3$P			-9697.574344	1.0255	-9697.583518	
PMe$_3$			-8111.575818	1.0180	-8111.575823	
PCy$_3$			-8697.354339	1.0263	-8697.354309	
P(OPh)$_3$			-8912.177300	1.0220	-8912.146136	
NMe$_3$			-7824.930570	1.0197	-7824.930542	
AsPh$_3$			-10580.900294	1.0194	-10580.90030	
SbPh$_3$			-8350.509685	1.0231	-8350.509650	
**Species**	**Hartrees**			**kcal/mol**		
	**H$_{corr}$ (sb)**	**G$_{corr}$ (sb)**	**E (lb)**	**G (lb)**	**G' (lb)**	**G'$_{rel}$ (lb)**
[NiBr(dppf)(L)] + PhCH$_2$CH$_2^·$ (corrected open shell singlet)						
dppf	1.264970	1.070158	-1908.693088	-6844636.7	-6844634.8	10.1
FcPPh$_2$	1.079279	0.907539	-10104.888405	-6340343.7	-6340341.8	7.2
PPh$_3$	1.004493	0.839665	-8686.463861	-5450311.5	-5450309.6	17.7
(*p*-MeOC$_6$H$_4$)$_3$P	1.110731	0.926256	-9029.966172	-5665808.1	-5665806.2	3.5
(*p*-MeC$_6$H$_4$)$_3$P	1.092924	0.913323	-8804.348048	-5524238.7	-5524236.8	18.9
(*p*-FC$_6$H$_4$)$_3$P	0.982193	0.808109	-8984.204523	-5637166.4	-5637164.5	4.5
(*p*-F$_3$CC$_6$H$_4$)$_3$P	1.029840	0.838297	-9697.564691	-6084787.7	-6084785.8	20.5
PMe$_3$	0.834511	0.690053	-8111.575812	-5089657.7	-5089655.8	5.9
PCy$_3$	1.221754	1.053422	-8697.354371	-5457011.2	-5457009.3	-0.2
P(OPh)$_3$	1.021195	0.853724	-8912.209866	-5591960.4	-5591958.5	-13.1
NMe$_3$	0.841687	0.702161	-7824.930599	-4909777.5	-4909775.6	15.1
AsPh$_3$	1.003726	0.837140	-10580.900288	-6639089.9	-6639088.0	9.4
SbPh$_3$	1.002795	0.837466	-8350.509722	-5239498.4	-5239496.6	11.2
**[NiBr(dppf)(L)] + PhCH$_2$CH$_2^·$ recombination TS**			**E (lb) (singlet)**	**<S**2>**	**E (lb) (triplet)**	
PMe$_3$ (tbp)[a]			-8111.567188	0.8703	-8111.558843	
PMe$_3$ (sqbp)[a]			-8111.576743	0.7320	-8111.553715	
PCy$_3$ (sqbp)[a]			-8697.327228	0.2767	-8697.324635	
PPh$_3$ (sqbp)[a]			-8686.458997	0.2708	-8686.434604	
**Species**	**Hartrees**			**kcal/mol**		
	**H$_{corr}$ (sb)**	**G$_{corr}$ (sb)**	**E (lb)**	**G (lb)**	**G' (lb)**	**G'$_{rel}$ (lb)**
**[NiBr(dppf)(L)] + PhCH$_2$CH$_2^·$ recombination TS (corrected open shell singlet)**						
PMe$_3$ (tbp)[a]	0.83496	0.698087	-8111.573617	-5089651.2	-5089649.3	12.3
PMe$_3$ (sqbp)[a]	0.835752	0.701997	-8111.590037	-5089659.1	-5089657.2	4.5
PCy$_3$ (sqbp)[a]	1.223390	1.061972	-8697.327644	-5456989.1	-5456987.2	31.7
PPh$_3$ (sqbp)[a]	1.007285	0.852523	-8686.462817	-54540302.7	-5450300.9	26.4
**[NiBr(CH$_2$CH$_2$Ph)(dppf)(L)]**						
PMe$_3$ (tbp)[a]	0.838149	0.704134	-8111.581880	-5089652.2	-5089650.3	11.4
PMe$_3$ (sqbp)[a]	0.838674	0.705403	-8111.594021	-5089659.5	-5089657.6	4.1
PCy$_3$ (sqbp)[a]	1.224167	1.060221	-8697.353012	-5457006.1	-5457004.2	14.6
PPh$_3$ (sqbp)[a]	1.009481	0.854085	-8686.489223	-5450318.3	-5450316.4	10.8
**[NiBr(CH$_2$CH$_2$Ph)(dppf)]**						
	0.713960	0.592008	-7650.562471	-4800428.9	-4800427.1	-4.2

[a] tbp = trigonal bipyramidal; sqbp = square-based pyramid.

5.5 Coordinates

Coordinates can be obtained in two ways:

- An XYZ file containing all coordinates from DFT calculations.
- From the IOChem-BD repository[29] at the DOI given in the manuscript.

6. References

(1) Yin, G.; Kalvet, I.; Englert, U.; Schoenebeck, F. Fundamental Studies and Development of Nickel-Catalyzed Trifluoromethylthiolation of Aryl Chlorides: Active Catalytic Species and Key Roles of Ligand and Traceless MeCN Additive Revealed. *J. Am. Chem. Soc.* **2015,** *137* (12), 4164.

(2) Standley, E. A.; Smith, S. J.; Müller, P.; Jamison, T. F. A Broadly Applicable Strategy for Entry into Homogeneous Nickel(0) Catalysts from Air-Stable Nickel(II) Complexes. *Organometallics* **2014,** *33* (8), 2012.

(3) Fulmer, G. R.; Miller, A. J. M.; Sherden, N. H.; Gottlieb, H. E.; Nudelman, A.; Stoltz, B. M.; Bercaw, J. E.; Goldberg, K. I. NMR Chemical Shifts of Trace Impurities: Common Laboratory Solvents, Organics, and Gases in Deuterated Solvents Relevant to the Organometallic Chemist. *Organometallics* **2010,** *29* (9), 2176.

(4) Liu, G.-B.; Zhao, H.-Y.; Dai, L.; Thiermann, T.; Tashiro, H.; Tashiro, M. Raney Ni-Al alloy-mediated reduction of benzils in water. *J. Chem. Res.* **2009,** *2009*, 579.

(5) Wagner, R. A.; Brinker, U. H. A Novel and Facile Synthesis of 2,3-Diphenylbuta-1,3-diene. *Synthesis* **2001,** *2001* (03), 0376.

(6) Prinsell, M. R.; Everson, D. A.; Weix, D. J. Nickel-catalyzed, sodium iodide-promoted reductive dimerization of alkyl halides, alkyl pseudohalides, and allylic acetates. *Chem. Commun.* **2010,** *46* (31), 5743.

(7) Sollott, G. P.; Mertwoy, H. E.; Portnoy, S.; Snead, J. L. Unsymmetrical Tertiary Phosphines of Ferrocene by Friedel-Crafts Reactions. I. Ferrocenylphenylphosphines. *J. Org. Chem.* **1963,** *28* (4), 1090.

(8) Frisch, M. J.; Trucks, G. W.; Schlegel, H. B.; Scuseria, G. E.; Robb, M. A.; Cheeseman, J. R.; Scalmani, G.; Barone, V.; Petersson, G. A.; Nakatsuji, H.et al. Wallingford, CT, 2016.

(9) Riplinger, C.; Neese, F. An efficient and near linear scaling pair natural orbital based local coupled cluster method. *J. Chem. Phys.* **2013,** *138* (3), 034106.

(10) Riplinger, C.; Sandhoefer, B.; Hansen, A.; Neese, F. Natural triple excitations in local coupled cluster calculations with pair natural orbitals. *J. Chem. Phys.* **2013,** *139* (13), 134101.

(11) Riplinger, C.; Pinski, P.; Becker, U.; Valeev, E. F.; Neese, F. Sparse maps—A systematic infrastructure for reduced-scaling electronic structure methods. II. Linear scaling domain based pair natural orbital coupled cluster theory. *J. Chem. Phys.* **2016,** *144* (2), 024109.

(12) Neese, F. The ORCA program system. *WIREs Computational Molecular Science* **2012,** *2* (1), 73.

(13) Neese, F. Software update: the ORCA program system, version 4.0. *WIREs Computational Molecular Science* **2018,** *8* (1), e1327.

(14) Becke, A. D. Density-functional thermochemistry. III. The role of exact exchange. *J. Chem. Phys.* **1993,** *98* (7), 5648.

(15) Lee, C.; Yang, W.; Parr, R. G. Development of the Colle-Salvetti correlation-energy formula into a functional of the electron density. *Phys. Rev. B.: Condens. Mater.* **1988,** *37* (2), 785.

(16) Vosko, S. H.; Wilk, L.; Nusair, M. Accurate spin-dependent electron liquid correlation energies for local spin density calculations: a critical analysis. *Canadian Journal of Physics* **1980,** *58* (8), 1200.

(17) Stephens, P. J.; Devlin, F. J.; Chabalowski, C. F.; Frisch, M. J. Ab Initio Calculation of Vibrational Absorption and Circular Dichroism Spectra Using Density Functional Force Fields. *J. Phys. Chem.* **1994,** *98* (45), 11623.

(18) Grimme, S.; Antony, J.; Ehrlich, S.; Krieg, H. A consistent and accurate ab initio parametrization of density functional dispersion correction (DFT-D) for the 94 elements H-Pu. *J. Chem. Phys.* **2010,** *132* (15), 154104.

(19) Ehlers, A. W.; Böhme, M.; Dapprich, S.; Gobbi, A.; Höllwarth, A.; Jonas, V.; Köhler, K. F.; Stegmann, R.; Veldkamp, A.; Frenking, G. A set of f-polarization functions for pseudo-

potential basis sets of the transition metals Sc□Cu, Y□Ag and La□Au. *Chemical Physics Letters* **1993,** *208* (1–2), 111.
(20) Hay, P. J.; Wadt, W. R. Ab initio effective core potentials for molecular calculations. Potentials for K to Au including the outermost core orbitals. *J. Chem. Phys.* **1985,** *82* (1), 299.
(21) Roy, L. E.; Hay, P. J.; Martin, R. L. Revised Basis Sets for the LANL Effective Core Potentials. *J. Chem. Theor. Comput.* **2008,** *4* (7), 1029.
(22) Zhao, Y.; Truhlar, D. G. Density Functionals with Broad Applicability in Chemistry. *Acc. Chem. Res.* **2008,** *41* (2), 157.
(23) Marenich, A. V.; Cramer, C. J.; Truhlar, D. G. Universal Solvation Model Based on Solute Electron Density and on a Continuum Model of the Solvent Defined by the Bulk Dielectric Constant and Atomic Surface Tensions. *J. Phys. Chem. B* **2009,** *113* (18), 6378.
(24) Harvey, J. N.; Himo, F.; Maseras, F.; Perrin, L. Scope and Challenge of Computational Methods for Studying Mechanism and Reactivity in Homogeneous Catalysis. *ACS Catal.* **2019,** *9* (8), 6803.
(25) Yamaguchi, K.; Jensen, F.; Dorigo, A.; Houk, K. N. A spin correction procedure for unrestricted Hartree-Fock and Møller-Plesset wavefunctions for singlet diradicals and polyradicals. *Chemical Physics Letters* **1988,** *149* (5), 537.
(26) Falivene, L.; Credendino, R.; Poater, A.; Petta, A.; Serra, L.; Oliva, R.; Scarano, V.; Cavallo, L. SambVca 2. A Web Tool for Analyzing Catalytic Pockets with Topographic Steric Maps. *Organometallics* **2016,** *35* (13), 2286.
(27) Poater, A.; Cosenza, B.; Correa, A.; Giudice, S.; Ragone, F.; Scarano, V.; Cavallo, L. SambVca: A Web Application for the Calculation of the Buried Volume of N-Heterocyclic Carbene Ligands. *Eur. J. Inorg. Chem.* **2009,** *2009* (13), 1759.
(28) Falivene, L.; Cao, Z.; Petta, A.; Serra, L.; Poater, A.; Oliva, R.; Scarano, V.; Cavallo, L. Towards the online computer-aided design of catalytic pockets. *Nat. Chem* **2019,** *11* (10), 872.
(29) Álvarez-Moreno, M.; de Graaf, C.; López, N.; Maseras, F.; Poblet, J. M.; Bo, C. Managing the Computational Chemistry Big Data Problem: The ioChem-BD Platform. *J. Chem. Inf. Model.* **2015,** *55* (1), 95.
